# Is there any room for ChatGPT AI bot in speech-language pathology?

**DOI:** 10.1007/s00405-025-09295-y

**Published:** 2025-03-01

**Authors:** Namık Yücel Birol, Hilal Berber Çiftci, Ayşegül Yılmaz, Ayhan Çağlayan, Ferhat Alkan

**Affiliations:** 1 Department of Speech and Language Therapy, Faculty of Health Sciences, Tarsus University, Mersin, Türkiye; 2 Department of Speech and Language Therapy, Graduate School of Health Sciences, İstanbul Medipol University, İstanbul, Türkiye; 3 Çağlayan Speech and Language Therapy Center, İzmir, Türkiye; 4 Department of Speech and Language Therapy, Institute of Graduate Education, İstinye University, İstanbul, Türkiye

**Keywords:** ChatGPT, Speech-language pathology, Artificial intelligence, Clinical support

## Abstract

**Purpose:**

This study investigates the potential of the ChatGPT-4.0 artificial intelligence bot to assist speech-language pathologists (SLPs) by assessing its accuracy, comprehensiveness, and relevance in various tasks related to speech, language, and swallowing disorders.

**Method:**

In this cross-sectional descriptive study, 15 practicing SLPs evaluated ChatGPT-4.0’s responses to task-specific queries across six core areas: report writing, assessment material generation, clinical decision support, therapy stimulus generation, therapy planning, and client/family training material generation. English prompts were created in seven areas: speech sound disorders, motor speech disorders, aphasia, stuttering, childhood language disorders, voice disorders, and swallowing disorders. These prompts were entered into ChatGPT-4.0, and its responses were evaluated. Using a three-point Likert-type scale, participants rated each response for accuracy, relevance, and comprehensiveness based on clinical expectations and their professional judgment.

**Results:**

The study revealed that ChatGPT-4.0 performed with predominantly high accuracy, comprehensiveness, and relevance in tasks related to speech and language disorders. High accuracy, comprehensiveness, and relevance levels were observed in report writing, clinical decision support, and creating education material. However, tasks such as creating therapy stimuli and therapy planning showed more variation with medium and high accuracy levels.

**Conclusions:**

ChatGPT-4.0 shows promise in assisting SLPs with various professional tasks, particularly report writing, clinical decision support, and education material creation. However, further research is needed to address its limitations in therapy stimulus generation and therapy planning to improve its usability in clinical practice. Integrating AI technologies such as ChatGPT could improve the efficiency and effectiveness of therapeutic processes in speech-language pathology.

**Supplementary Information:**

The online version contains supplementary material available at 10.1007/s00405-025-09295-y.

## Introduction

ChatGPT (Chat Generative Pre-Trained Transformer) is an AI-powered chatbot designed to chat with users like humans [1]. The data obtained with ChatGPT covers a wide range of internet resources such as books, articles and websites. ChatGPT mimics human language using an internet-based database to process entered text and generate responses based on entered prompts. ChatGPT aims to generate text that mimics natural human language, which can be used for various processing tasks such as language translation, text summarisation and dialogue systems. In addition, chatbots can be used for tasks such as generating responses, answering questions and writing creative stories [2]. Natural language processing models, an area of artificial intelligence, can be a valuable tool for personalised approaches. Using deep learning, ChatGPT has been used in many sectors, including marketing, education, engineering and healthcare. One of ChatGPT’s capabilities is its ability to filter large amounts of information and provide answers in a conversational and easy-to-understand manner.

Integrating artificial intelligence (AI) into healthcare has received a growing interest, offering potential improvements in diagnosis, treatment and patient management [3]. ChatGPT can significantly impact the diagnosis of diseases by improving accuracy, speed and efficiency of decision-making procedures [4]. Advances in technology have meant that patients now turn not only to healthcare professionals for medical information but also to search engines and, more recently, AI chatbots [5]. A recent study showed that peer-to-peer mental health was supported with HAILEY - an AI model that can rewrite the given text more empathetically [6]. Another study found that ChatGPT has the potential to automate the process of documenting patient data in nursing practices and answering clinical questions of nurses [7]. When examined in the field of diagnosis and triage in medicine, it was found that Generative Pre-trained Transformer 3 (GPT-3), an artificial intelligence application, was better than non-physicians, and close to, but less successful than physicians [8]. ChatGPT-3.5 offers improved workflow to support clinical decision making in radiology [9]. In a previous study on the treatment of unilateral vocal fold paralysis (UVFP), 20 clinical cases were presented to ChatGPT-4.0 and Llama (Large Language Model Meta AI) Chat-2.0 to request potential treatment strategies [10]. The results of this study indicated that although ChatGPT significantly outperformed Llama, it may have harmful consequences, such as recommending medialization for patients with stridor and shortness of breath and it was shown in the study that making treatment decisions for complex conditions such as UVFP is beyond the capabilities of ChatGPT. In Obstetrics and Gynaecology, clinicians have evaluated the ability of ChatGPT-3.5 to address/perceive and respond to questions [11]. In that study, they found that ChatGPT was meaningful and informative for almost every topic but needed improvements, particularly on account of outdated databases, inability to cite sources, and inability to understand what the user really wanted. While hinting at potential advantages of incorporating AI in healthcare, the previous studies also call for more detailed studies in different health-related disciplines to better gauge usability of AI tools to improve health outcomes and therapeutic processes.

Through the algorithm in ChatGPT, the outputs received for each case are explicitly tailored to the needs. ChatGPT can summarise clinically entered patient data, so case data can be collected more systematically and quickly [1]. There is a view that ChatGPT, which can generate diagnostic and therapeutic outcome reports based on the patient’s current information, needs to be regularly updated and trained. Given that there may be biased or inaccurate information in its database, which is based on the Internet, and that ChatGPT uses this database containing this information, this can be seen as a limitation. OpenAI publicly released ChatGPT-3.5 in November 2020, and the newer version, ChatGPT-4.0, was made available for a fee on 14 March 2023. Recent studies evaluating AI in healthcare have compared ChatGPT-3.5 and ChatGPT-4.0 and found that the new version 4.0 was able to produce better responses in terms of accuracy, clarity and comprehensiveness [12, 13].

Using language models to positively impact health and improve patients’ lives is inevitable, but the impact and risks still need to be discovered [14]. One area of interest is the application of AI in speech-language pathology (SLP), a field that has traditionally relied on human expertise to diagnose and treat communication disorders. The potential positive impact of ChatGPT on clinical documentation and health communication has been highlighted by the suggestion that ChatGPT could be integrated into the training of SLP students to improve their plain language writing skills [15]. It still needs to be determined whether ChatGPT can be an effective tool for assessment and intervention in the field of SLP. Given that SLP is a complex and challenging field requiring extensive training to address communication disorders, professionals must perform detailed examinations to achieve acceptable performance in clinical practice [16]. However, incorporating AI technologies into assessment and intervention programmes alongside traditional speech and language therapy approaches can improve outcomes by enhancing patient care [17]. Despite the increasing adoption of web and mobile technology tools during therapy among speech-language pathologists (SLPs), there is a growing need and knowledge gap on applying natural language processing tools to improve efficiency [18]. In the field of SLP, ChatGPT-3.5 was tested on real speech samples from people with aphasia using the AphasiaBank. In this study, ChatGPT was found to be 91.67% accurate in detecting the target word and successful in combining politeness strategies [19]. Another study on aphasia investigated the potential use of DALL-E, an artificial intelligence image generation tool, in aphasia [20]. This study found that 189 of the 200 target images generated were successful, but it was also noted that there were aesthetic flaws that could affect the usefulness of the images. The potential for clinical functioning for receptive and expressive language, one of the areas of SLP that ASHA refers to as the ‘Big 9’ [21], was assessed using ChatGPT [22]. When the responses given by ChatGPT to the language intervention activities proposed in this study were examined, it was predicted that it may have the potential to be an innovative tool for SLPs in clinical practice. Recent studies have begun exploring how AI tools like ChatGPT can support SLP practice, providing a range of benefits while also highlighting areas of concern and limitations. Austin et al. (2024) examined the perceptions of SLP students and clinicians, who expressed hesitation about adopting AI tools due to concerns about their reliability and ethical implications [23]. The study noted that while ChatGPT and similar technologies could reduce clinical workloads by assisting with documentation and material creation, there is a need for clear institutional guidelines and training to encourage safe and effective use​. Similarly, Suh et al. (2024) investigated the integration of AI into SLP and found that early adopters recognised ChatGPT’s potential for producing materials such as assessment templates and support strategies for a range of disorders, including articulation and language issues [24]. The findings indicate that AI could streamline tasks, such as creating stimulus materials, but underscore the need for further research to address bias and ethical concerns, as well as to understand better how these tools could complement traditional SLP methods without replacing essential human oversight​. Together, these studies suggest that AI, while promising, must be carefully integrated into clinical practice. ChatGPT’s potential to complement SLP methods—particularly in areas like documentation and therapy planning—is balanced by limitations in language-specific nuances and ethical considerations, highlighting the need for comprehensive research and practitioner guidance.

It remains to be seen whether ChatGPT can support SLPs’ clinical services in assessment, clinical decision-making, production of therapy materials and mimicking humanoid communication. Evidence suggests that SLPs need a comprehensive and systematic review of data for clinical practice. There is a natural tendency to trust AI chatbot applications because of their similarity to human behaviour and ability to mimic responses. There is a need to demonstrate the usability of artificial intelligence bots in the field of SLP, particularly in personalised assessment and intervention.

By providing a comprehensive patient-specific analysis of the potential benefits and drawbacks of using ChatGPT in the field of SLP, we aim to inform healthcare providers and decision-makers about the potential of this technology to improve patient care. In summary, this study addresses the critical question: “Can ChatGPT 4.0 effectively support speech-language pathologists in their professional tasks?”.

## Method

This cross-sectional descriptive study aims to investigate the accuracy, comprehensiveness and relevance of the ChatGPT-4.0 artificial intelligence bot’s responses to various tasks related to speech, language and swallowing disorders.

### Procedure

Six tasks were identified for the usability of the ChatGPT-4 artificial intelligence bot in the field of SLP. These tasks are report writing, assessment material generation, clinical decision support, therapy stimulus generation, therapy planning and client/family training material generation. Based on the most common disorders associated with the tasks, English prompts were created in 7 areas: speech sound disorders, motor speech disorders, aphasia, stuttering, childhood speech disorders, voice disorders and swallowing disorders. These prompts and the tasks examined in the study were created by the authors of this article, five SLPs who are PhD students, by consensus in three online panels. The tasks and the disorders to be analysed were determined in the first panel. In the other two panels, prompts and hypothetical cases in the prompts were created.

The generated prompts were entered into the ChatGPT-4.0 artificial intelligence bot by the first author of the article between July 10 and 11, 2023 and the responses generated by ChatGPT-4 were recorded. In order to evaluate the accuracy, comprehensiveness and relevance parameters of the responses generated by ChatGPT-4, a three-point Likert-type questionnaire with low (1), medium (2) and high (3) responses was created. Accuracy refers to the factual correctness and clinical soundness of ChatGPT’s responses, assessing whether the information provided aligns with evidence-based practices and accepted clinical standards in SLP. Comprehensiveness measures the extent to which ChatGPT’s responses include all necessary details required to complete each task effectively, ensuring that no critical information is omitted. Lastly, relevance evaluates whether ChatGPT’s responses are appropriate and directly applicable to the specific clinical scenario, avoiding unnecessary or extraneous information that could detract from the task’s focus. Together, these parameters provide a thorough framework for assessing the quality and clinical utility of ChatGPT’s output in SLP contexts.

The evaluation criteria were designed based on standard clinical expectations for content quality, ensuring that responses would meet professional standards typically expected in SLP practice. ChatGPT’s performance was indirectly compared to clinical knowledge and expectations commonly held in the field of Speech-Language Pathology. While we did not use a specific gold standard text or tool, the participants, who are practicing clinicians, applied their professional judgment and knowledge as benchmarks when assessing ChatGPT’s responses. This approach ensured that the AI outputs were evaluated against real-world clinical expectations.

To ensure consistency, participants received a written guideline document detailing the evaluation criteria and expectations for each SLP task. This guideline outlined the typical content and standards expected in each task, providing a framework for rating accuracy, comprehensiveness, and relevance. This guideline did not dictate specific answers but provided a reference for assessing whether ChatGPT’s responses met clinical expectations in SLP.

In addition, a demographic information form was prepared to obtain information about the participants’ level of education, experience and employment sector. The prompts entered into ChatGPT-4, the responses generated, the demographic information form (education level, experience, sector of employment) and the related questionnaire were made online via Google Forms ***(Supplementary Information)***. Approval was obtained from the Non-Interventional Clinical Ethics Committee of Cappadocia University on April 14 2023, with the decision of the ethics committee numbered E-64577500-050.99-44211, stating that there was no ethical problem in conducting this research. All participants were informed about this study in detail and were enrolled if they agreed to participate.

### Participants

The relevant questionnaire was distributed via social networks, and SLPs, at least at the graduate student level, were asked to complete it. The questionnaire took approximately 150 min to complete. A total of 15 participants (4 master’s students and 11 doctoral students) completed the questionnaire. All participants were also practising clinicians with active experience in clinical settings. This dual role provided a unique perspective, as participants were able to assess ChatGPT’s applicability from both an academic and a practical, hands-on viewpoint.

### Statistical analysis of data

The data from our study were analysed using the IBM SPSS 27 statistical analysis software. Frequency, percentage, mean, median, standard deviation and minimum-maximum values were calculated for the Likert-type ratings given by the participants on different tasks in different disorders. Friedman test was used for statistical comparison of accuracy, comprehensiveness and relevance parameters in different tasks in different disorders. For inferential statistics, non-parametric tests were used as the data were Likert-type (Mircioiu & Atkinson, 2017). When there was significance, Bonferroni corrected Wilcoxon test was performed to determine which tasks were significant. A value of *p* < 0.05 in the Friedman test and *p* < 0.0033 in the Wilcoxon test with Bonferroni correction was considered significant. The inter-rater reliability was assessed using a two-way mixed-effects model with absolute agreement. The average measures intraclass correlation coefficient (ICC) was 0.755, 95% confidence interval (CI) [0.677, 0.818], indicating good reliability.

## Results

This study included 15 participants with varying levels of education and work experience. The demographic distribution is shown in Table 1.

**Table 1:** Participants demographics

	*n (%)*
**Education Level**	
Bachelor’s degree	4 (26.7%)
Master’s degree	11 (73.3%)
**Work Experience**	
1–3 years	4 (26.7%)
3–5 years	9 (60.0%)
5–9 years	2 (13.3%)
**Work Sector**	
Academia	8 (53.3%)
Hospital	5 (33.3%)
Private clinic	4 (26.7%)
Special education and rehabilitation center	1 (6.7%)

Table 2 shows the accuracy ratings for ChatGPT-4.0’s responses across different tasks and disorders. For speech sound disorders, accuracy was predominantly high for report writing, clinical decision-making, and creating education material. However, accuracy was lower for creating assessment material and therapy stimuli. For example, when tasked with generating minimal pairs involving the sounds /p/ and /b/, ChatGPT incorrectly provided “pati” (paw) and “bati” (a non-existent word in Turkish) as minimal pairs. This inaccuracy, stemming from a lack of linguistic knowledge in Turkish, impacted the accuracy rating, as the tool generated a word that does not have a meaningful equivalent. Additionally, in creating sentence-level articulation material, ChatGPT produced nonsensical phrases such as “Ğaileli ğuldu, ğildan yana dön döndü,” which incorrectly used the Turkish letter “ğ” in initial positions, a phonologic impossibility in Turkish grammar. These errors highlight ChatGPT’s limitations in language-specific nuances and led to low accuracy scores in this category. For motor speech disorders, accuracy was high across all tasks. For aphasia, accuracy was also generally high, except for a moderate rating for creating assessment material. In childhood language disorders, accuracy was high for all tasks except moderate ratings for creating assessment material and therapy stimuli. For stuttering, accuracy ranged from moderate to high across tasks. Voice disorders and swallowing disorders showed high accuracy ratings for all tasks, with a few moderate ratings for generating therapy stimuli.

**Table 2:** Accuracy findings on different tasks in different disorders

		Report writing	Creating assessment material	Clinical decision support	Creating therapy stimuli	Therapy planning	Creating education material
		n (%)	n (%)	n (%)	n (%)	n (%)	n (%)
SSD	Low	-	4 (26.7)	-	6 (40%)	-	-
	Medium	3 (20%)	10 (66.7%)	1 (6.7%)	9 (60%)	7 (46.7%)	5 (33.3%)
	High	12 (80%)	1 (6.7%)	14 (93.3%)	-	8 (53.3%)	10 (66.7%)
MSD	Low	-	-	-	-	-	-
	Medium	2 (13.3%)	2 (13.3%)	1 (6.7%)	3 (20%)	2 (13.3%)	3 (20%)
	High	13 (86.7%)	13 (86.7%)	14 (93.3%)	12 (80%)	13 (86.7%)	12 (80%)
Aphasia	Low	-	-	-	-	-	-
	Medium	2 (13.3%)	1 (6.7%)	2 (13.3%)	3 (20%)	2 (13.3%)	1 (6.7%)
	High	13 (86.7%)	14 (93.3%)	13 (86.7%)	12 (80%)	13 (86.7%)	14 (93.3%)
Stuttering	Low	-	-	-	3 (20%)	-	-
	Medium	2 (13.3%)	2 (13.3%)	3 (20%)	7 (46.7%)	3 (20%)	3 (20%)
	High	13 (86.7%)	13 (86.7%)	12 (80%)	5 (33.3%)	12 (80%)	12 (80%)
CLD	Low	-	-	-	-	-	-
	Medium	3 (20%)	4 (26.7%)	3 (20%)	2 (13.3%)	3 (20%)	1 (6.7%)
	High	12 (80%)	11 (73.3%)	12 (80%)	13 (86.7%)	12 (80%)	14 (93.3%)
Voice	Low	-	-	-	-	-	-
	Medium	1 (6.7%)	4 (26.7%)	2 (13.3%)	7 (46.7%)	1 (6.7%)	2 (13.3%)
	High	14 (93.3%)	11 (73.3%)	13 (86.7%)	8 (53.3%)	14 (93.3%)	13 (86.7%)
Swallowing	Low	-	-	-		-	-
	Medium	3 (20%)	4 (26.7%)	4 (26.7%)		3 (20%)	2 (13.3%)
	High	12 (80%)	11 (73.3%)	11 (73.3%)		12 (80%)	13 (86.7%)

*SSD: Speech Sound Disorders, MSD: Motor Speech Disorders, CLD: Childhood Language Disorders*

ChatGPT’s accuracy was assessed across tasks and disorder categories (Table 3), including report writing, assessment material creation, clinical decision support, therapy stimuli creation, therapy planning, and educational material creation. Mean accuracy scores ranged from 1.60 to 2.93 on a 1–3 scale. In Speech Sound Disorders, significant differences were observed (*p* < 0.001), with report writing (M = 2.80) and clinical decision support (M = 2.93) scoring highest, while creating therapy stimuli scored lowest (M = 1.60). Post hoc analysis showed significant differences between several task pairs for Speech Sound Disorders (*p* < 0.001). For Stuttering, report writing and assessment material creation were highest in accuracy (M = 2.87), with therapy stimuli creation lowest (M = 2.13) and notable task differences (*p* = 0.003). Overall, accuracy across most tasks and disorder categories was high.

**Table 3:** Comparison of the accuracy of different tasks in different disorders

	Report writing	Creating assessment material	Clinical decision support	Creating therapy stimuli	Therapy planning	Creating education material	*p*	Post hoc
	M ± SD Mdn (Min-Max)	M ± SD Mdn (Min-Max)	M ± SD Mdn (Min-Max)	M ± SD Mdn (Min-Max)	M ± SD Mdn (Min-Max)	M ± SD Mdn (Min-Max)		
SSD	2.80 ± 0.41 3.00 (2–3)	1.80 ± 0.56 2.00 (1–3)	2.93 ± 0.26 3.00 (2–3)	1.60 ± 0.51 2.00 (1–2)	2.53 ± 0.51 3.00 (2–3)	2.67 ± 0.49 3.00 (2–3)	< 0.001*	RW-CAM, RW-CTS, CAM-CDS, CDS-CTS, CTS-CEM
MSD	2.87 ± 0.35 3.00 (2–3)	2.87 ± 0.35 3.00 (2–3)	2.93 ± 0.26 3.00 (2–3)	2.80 ± 0.41 3.00 (2–3)	2.87 ± 0.35 3.00 (2–3)	2.80 ± 0.41 3.00 (2–3)	0.765	-
Aphasia	2.87 ± 0.35 3.00 (2–3)	2.93 ± 0.26 3.00 (2–3)	2.87 ± 0.35 3.00 (2–3)	2.80 ± 0.41 3.00 (2–3)	2.87 ± 0.35 3.00 (2–3)	2.93 ± 0.26 3.00 (2–3)	0.594	-
Stuttering	2.87 ± 0.35 3.00 (2–3)	2.87 ± 0.35 3.00 (2–3)	2.80 ± 0.41 3.00 (2–3)	2.13 ± 0.74 2.00 (1–3)	2.80 ± 0.41 3.00 (2–3)	2.80 ± 0.41 3.00 (2–3)	< 0.001*	-
CLD	2.80 ± 0.41 3.00 (2–3)	2.73 ± 0.46 3.00 (2–3)	2.80 ± 0.41 3.00 (2–3)	2.87 ± 0.35 3.00 (2–3)	2.80 ± 0.41 3.00 (2–3)	2.93 ± 0.26 3.00 (2–3)	0.577	-
Voice	2.93 ± 0.26 3.00 (2–3)	2.73 ± 0.46 3.00 (2–3)	2.87 ± 0.35 3.00 (2–3)	2.53 ± 0.51 3.00 (2–3)	2.93 ± 0.26 3.00 (2–3)	2.87 ± 0.35 3.00 (2–3)	< 0.001*	-
Swallowing	2.80 ± 0.41 3.00 (2–3)	2.73 ± 0.46 3.00 (2–3)	2.73 ± 0.46 3.00 (2–3)	-	2.80 ± 0.41 3.00 (2–3)	2.87 ± 0.35 3.00 (2–3)	0.539	-

SSD: Speech Sound Disorders, MSD: Motor Speech Disorders, CLD: Childhood Language Disorders, RW: Report Writing, CAM: Creating Assessment Material, CDS: Clinical Decision Support, CTS: Creating Therapy Stimuli, TP: Therapy Planning, CEM: Creating Education Material, *p* < 0.05, Bonferroni corrected *p* < 0,0033*

Table 4 displays the comprehensiveness ratings. For speech sound disorders, comprehensiveness was high for clinical decision-making and therapy planning, but lower for creating assessment material and stimuli. For example, in generating educational materials for Childhood Apraxia of Speech (CAS), ChatGPT included key components like CAS diagnosis, common symptoms, and home support strategies, earning a high comprehensiveness rating. However, it lacked additional practical resources, such as visual aids, which may have lowered comprehensiveness slightly. Motor speech disorders and aphasia showed moderate to high comprehensiveness. For childhood language disorders and stuttering, ratings ranged from moderate to high. Voice disorders had moderate to high comprehensiveness, while swallowing disorders ranged from moderate to high comprehensiveness.

**Table 4:** Comprehensiveness findings on different tasks in different disorders

		Report writing	Creating assessment material	Clinical decision support	Creating therapy stimuli	Therapy planning	Creating education material
		n (%)	n (%)	n (%)	n (%)	n (%)	n (%)
SSD	Low	-	1 (6.7%)	1 (6.7%)	3 (20%)	-	-
	Medium	6 (40%)	12 (80.0%)	3 (20%)	8 (53.3%)	4 (26.7%)	8 (53.3%)
	High	9 (60%)	2 (13.3%)	11 (73.3%)	4 (26.7%)	11 (73.3%)	7 (46.7%)
MSD	Low	-	-	-	-	-	1 (6.7%)
	Medium	4 (26.7%)	5 (33.3%)	1 (6.7%)	5 (33.3%)	5 (33.3%)	4 (26.7%)
	High	11 (73.3%)	10 (66.7%)	14 (93.3%)	10 (66.7%)	10 (66.7%)	10 (66.7%)
Aphasia	Low	-	1 (6.7%)	-	-	-	-
	Medium	3 (20%)	5 (33.3%)	2 (13.3%)	6 (40%)	7 (46.7%)	7 (46.7%)
	High	12 (80%)	9 (60%)	13 (86.7%)	9 (60%)	8 (53.3%)	8 (53.3%)
Stuttering	Low	-	1 (6.7%)	-	1 (6.7%)	-	-
	Medium	4 (26.7%)	3 (20%)	8 (53.3%)	9 (60%)	7 (46.7%)	4 (26.7%)
	High	11 (73.3%)	11 (73.3%)	7 (46.7%)	5 (33.3%)	8 (53.3%)	11 (73.3%)
CLD	Low	-	-	-	-	-	-
	Medium	5 (33.3%)	9 (60%)	8 (53.3%)	5 (33.3%)	9 (60%)	4 (26.7%)
	High	10 (66.7%)	6 (40%)	7 (46.7%)	10 (66.7%)	6 (40%)	11 (73.3%)
Voice	Low	-	-	-	-	-	-
	Medium	3 (20%)	5 (33.3%)	4 (26.7%)	7 (46.7%)	3 (20%)	4 (26.7%)
	High	12 (80%)	10 (66.7%)	11 (73.3%)	8 (53.3%)	12 (80%)	11 (73.3%)
Swallowing	Low	-	-	-		-	-
	Medium	5 (33.3%)	4 (26.7%)	6 (40%)		5 (33.3%)	3 (20%)
	High	10 (66.7%)	11 (73.3%)	9 (60%)		10 (66.7%)	12 (80%)

*SSD: Speech Sound Disorders, MSD: Motor Speech Disorders, CLD: Childhood Language Disorders*

Table 5 presents comprehensiveness scores, with means ranging from 2.07 to 2.93. Significant differences were observed in Speech Sound Disorders (*p* < 0.001), with therapy planning scoring highest (M = 2.73) and assessment material creation scoring lowest (M = 2.07). Post hoc analysis indicated significant differences between several task pairs within Speech Sound Disorders. Overall, comprehensiveness across most tasks and disorder categories was high.

**Table 5:** Comparison of the comprehensiveness of different tasks in different disorders

	Report writing	Creating assessment material	Clinical decision support	Creating therapy stimuli	Therapy planning	Creating education material	*p*	Post hoc
	M ± SD Mdn (Min-Max)	M ± SD Mdn (Min-Max)	M ± SD Mdn (Min-Max)	M ± SD Mdn (Min-Max)	M ± SD Mdn (Min-Max)	M ± SD Mdn (Min-Max)		
SSD	2.60 ± 0.50	2.07 ± 0.46	2.67 ± 0.62	2.07 ± 0.70	2.73 ± 0.46	2.47 ± 0.51	< 0.001*	CAM-
	3.00	2.00	3.00	2.00	3.00	2.00		CDS, CAM-TP
	(2–3)	(1–3)	(1–3)	(1–3)	(2–3)	(2–3)		
MSD	2.73 ± 0.46	2.67 ± 0.49	2.93 ± 0.26	2.67 ± 0.49	2.67 ± 0.49	2.60 ± 0.63	0.297	-
	3.00	3.00	3.00	3.00	3.00	3.00		
	(2–3)	(2–3)	(2–3)	(2–3)	(2–3)	(1–3)		
Aphasia	2.80 ± 0.41	2.53 ± 0.64	2.87 ± 0.35	2.60 ± 0.51	2.53 ± 0.51	2.53 ± 0.51	0.082	-
	3.00	3.00	3.00	3.00	3.00	3.00		
	(2–3)	(1–3)	(2–3)	(2–3)	(2–3)	(2–3)		
Stuttering	2.73 ± 0.49	2.67 ± 0.62	2.47 ± 0.51	2.27 ± 0.59	2.53 ± 0.51	2.73 ± 0.46	0.033*	-
	3.00	3.00	2.00	2.00	3.00	3.00		
	(2–3)	(1–3)	(2–3)	(1–3)	(2–3)	(2–3)		
CLD	2.67 ± 0.49	2.40 ± 0.51	2.47 ± 0.51	2.67 ± 0.49	2.40 ± 0.51	2.73 ± 0.46	0.099	-
	3.00	2.00	2.00	3.00	2.00	3.00		
	(2–3)	(2–3)	(2–3)	(2–3)	(2–3)	(2–3)		
Voice	2.80 ± 0.41	2.67 ± 0.49	2.73 ± 0.46	2.53 ± 0.51	2.80 ± 0.41	2.73 ± 0.46	0.320	-
	3.00	3.00	3.00	3.00	3.00	3.00		
	(2–3)	(2–3)	(2–3)	(2–3)	(2–3)	(2–3)		
Swallowing	2.67 ± 0.49	2.73 ± 0.46	2.60 ± 0.51	-	2.67 ± 0.49	2.80 ± 0.41	0.627	-
	3.00	3.00	3.00		3.00	3.00		
	(2–3)	(2–3)	(2–3)		(2–3)	(2–3)		

SSD: Speech Sound Disorders, MSD: Motor Speech Disorders, CLD: Childhood Language Disorders, RW: Report Writing, CAM: Creating Assessment Material, CDS: Clinical Decision Support, CTS: Creating Therapy Stimuli, TP: Therapy Planning, CEM: Creating Education Material, *p* < 0.05*, Bonferroni corrected *p* < 0,0033*

Table 6 shows the relevance ratings. For most disorders, relevance was rated as predominantly high, except for some moderate ratings. For instance, in a therapy planning task involving a client with hypokinetic dysarthria, ChatGPT’s response included suggestions for the Lee Silverman Voice Treatment (LSVT LOUD^®^), a common and effective approach for Parkinson’s-related speech issues. This recommendation was highly relevant, as it aligned with widely accepted treatment strategies, thus supporting the therapeutic goals effectively. The response’s relevance was further enhanced by ChatGPT’s ability to outline key elements of the therapy, reinforcing its appropriateness for motor speech disorder management. Speech sound disorders had lower relevance for assessment/stimuli generation compared to other tasks.

**Table 6:** Relevance findings on different tasks in different disorders

		Report writing	Creating assessment material	Clinical decision support	Creating therapy stimuli	Therapy planning	Creating education material
		n (%)	n (%)	n (%)	n (%)	n (%)	n (%)
SSD	Low	-	-	-	2 (13.3%)	-	-
	Medium	1 (6.7%)	7 (46.7%)	2 (13.3%)	5 (33.3%)	3 (20%)	3 (20%)
	High	14 (93.3%)	8 (53.3%)	13 (86.7%)	8 (53.3%)	12 (80%)	12 (80%)
MSD	Low	-	-	-	-	-	1 (6.7%)
	Medium	2 (13.3%)	3 (20%)	1 (6.7%)	4 (26.7%)	2 (13.3%)	3 (20%)
	High	13 (86.7%)	12 (80%)	14 (93.3%)	11 (73.3%)	13 (86.7%)	11 (73.3%)
Aphasia	Low	-	-	-	-	-	-
	Medium	2 (13.3%)	2 (13.3%)	2 (13.3%)	3 (20%)	1 (6.7%)	3 (20%)
	High	13 (86.7%)	13 (86.7%)	13 (86.7%)	12 (80%)	14 (93.3%)	12 (80%)
Stuttering	Low	-	-	-	-	-	-
	Medium	3 (20%)	2 (13.3%)	4 (26.7%)	6 (40%)	2 (13.3%)	2 (13.3%)
	High	12 (80%)	13 (86.7%)	11 (73.3%)	9 (60%)	13 (86.7%)	13 (86.7%)
CLD	Low	-	-	-	-	-	-
	Medium	1 (6.7%)	3 (20%)	3 (20%)	1 (6.7%)	1 (6.7%)	1 (6.7%)
	High	14 (93.3%)	12 (80%)	12 (80%)	14 (93.3%)	14 (93.3%)	14 (93.3%)
Voice	Low	-	-	-	-	-	-
	Medium	1 (6.7%)	4 (26.7%)	1 (6.7%)	2 (13.3%)	1 (6.7%)	1 (6.7%)
	High	14 (93.3%)	11 (73.3%)	14 (93.3%)	13 (86.7%)	14 (93.3%)	14 (93.3%)
Swallowing	Low	-	-	-		-	-
	Medium	3 (20%)	2 (13.3%)	4 (26.7%)		2 (13.3%)	2 (13.3%)
	High	12 (80%)	13 (86.7%)	11 (73.3%)		13 (86.7%)	13 (86.7%)

*SSD: Speech Sound Disorders, MSD: Motor Speech Disorders, CLD: Childhood Language Disorders*

The relevance of ChatGPT’s outputs was measured and presented in Table 7. The relevance scores were high across most tasks, indicating that ChatGPT provides outputs that are pertinent to the tasks at hand. The scores for relevance varied, with means ranging from 2.40 to 2.93.

**Table 7:** Comparison of the relevance of different tasks in different disorders

	Report writing	Creating assessment material	Clinical decision support	Creating therapy stimuli	Therapy planning	Creating education material	*p*	Post hoc
	M ± SD Mdn (Min-Max)	M ± SD Mdn (Min-Max)	M ± SD Mdn (Min-Max)	M ± SD Mdn (Min-Max)	M ± SD Mdn (Min-Max)	M ± SD Mdn (Min-Max)		
SSD	2.93 ± 0.	2.53 ± 0.51	2.87 ± 0.3	2.40 ± 0.7	2.80 ± 0.4	2.80 ± 0.41	0.002*	-
	26	3.00	5	4	1	3.00		
	3.00	(2–3)	3.00	3.00	3.00	(2–3)		
	(2–3)		(2–3)	(1–3)	(2–3)			
MSD	2.87 ± 0.	2.80 ± 0.41	2.93 ± 0.2	2.73 ± 0.4	2.87 ± 0.3	2.67 ± 0.0.6	0.333	-
	35	3.00	6	6	5	2		
	3.00	(2–3)	3.00	3.00	3.00	3.00		
	(2–3)		(2–3)	(2–3)	(2–3)	(1–3)		
Aphasia	2.87 ± 0.	2.87 ± 0.35	2.87 ± 0.3	2.80 ± 0.4	2.93 ± 0.2	2.80 ± 0.41	0.340	-
	35	3.00	5	1	6	3.00		
	3.00	(2–3)	3.00	3.00	3.00	(2–3)		
	(2–3)		(2–3)	(2–3)	(2–3)			
Stuttering	2.80 ± 0.	2.87 ± 0.35	2.73 ± 0.4	2.60 ± 0.5	2.87 ± 0.3	2.87 ± 0.35	0.128	-
	41	3.00	9	1	5	3.00		
	3.00	(2–3)	3.00	3.00	3.00	(2–3)		
	(2–3)		(2–3)	(2–3)	(2–3)			
CLD	2.93 ± 0.	2.80 ± 0.41	2.80 ± 0.4	2.93 ± 0.2	2.93 ± 0.2	2.93 ± 0.26	0.075	-
	26	3.00	1	6	6	3.00		
	3.00	(2–3)	3.00	3.00	3.00	(2–3)		
	(2–3)		(2–3)	(2–3)	(2–3)			
Voice	2.93 ± 0.	2.73 ± 0.46	2.93 ± 0.2	2.87 ± 0.3	2.93 ± 0.2	2.93 ± 0.26	0.032*	-
	26	3.00	6	5	6	3.00		
	3.00	(2–3)	3.00	3.00	3.00	(2–3)		
	(2–3)		(2–3)	(2–3)	(2–3)			
Swallowing	2.80 ± 0.	2.87 ± 0.35	2.73 ± 0.4	-	2.87 ± 0.3	2.87 ± 0.0.3	0.255	-
	41	3.00	6		5	5		
	3.00	(2–3)	3.00		3.00	3.00		
	(2–3)		(2–3)		(2–3)	(2–3)		

SSD: Speech Sound Disorders, MSD: Motor Speech Disorders, CLD: Childhood Language Disorders, RW: Report Writing, CAM: Creating Assessment Material, CDS: Clinical Decision Support, CTS: Creating Therapy Stimuli, TP: Therapy Planning, CEM: Creating Education Material, *p* < 0.05*, Bonferroni corrected *p* < 0,0033*

## Discussion

The debate on whether AI chatbots will replace humans or assist humans in their professions is still ongoing. In this study, we investigated the potential use of ChatGPT in the field of SLP, which signals the possibility of its use in different fields.

According to the results of our research, when the performance of ChatGPT 4.0 in the field of SLP was analysed according to different tasks and disorders. ChatGPT 4.0 generally showed high accuracy, comprehensiveness and relevance. ChatGPT 4.0 performed particularly strongly on report writing and clinical decision-making tasks. While some areas were associated with lower performance, such as generating therapy stimuli for specific disorders, the overall findings suggest that ChatGPT 4.0 can be a valuable tool in supporting SLP tasks. This aligns with findings from [24], who identified key areas where AI-based tools can enhance the capacity and job satisfaction of SLPs by addressing their needs, constraints, and challenges.

In this study, when the accuracy of the responses given by ChatGPT 4.0 to different tasks belonging to different disorders was examined, it was found that the responses in the areas evaluated in most disorders were at a high level in accuracy. In the tasks related to SSD, a significant difference was found between creating assessment material and, respectively; report writing-clinical decision making-therapy planning. A significant difference was also found between creating therapy stimuli and, respectively; report writing-clinical decision making and creating educational material. It was observed that the performance in the tasks of creating assessment material and creating therapy stimuli was lower than the other tasks. ChatGPT 4.0 may have been weak in this area since it was asked to create Turkish-specific materials in these tasks, given that ChatGPT 4.0’s effectiveness is conditioned upon the quality and scope of language-specific training data. In specialised fields such as Speech-Language Pathology in Turkish, the results may lack sufficient depth, particularly in regard to specialised terminology and culturally relevant stimuli. These deficiencies contribute to responses that are more generalised or less accurate in Turkish compared to those generated in languages supported by more extensive datasets, such as English [25]. It was found that ChatGPT 4.0 shows lower performance in creating therapy stimuli in stuttering tasks. In a study examining the relationship between speech therapy and artificial intelligence, ChatGPT 3.5 and ChatGPT 4.0 were used to create therapy stimuli and materials [26]. In this study, when ChatGPT was used for a simple task such as creating a story involving phonemic awareness, the results of this task were positive. When the age of the child was changed and the situation was complicated by including additional disorders, it was reported that the responses had to be edited. Based on these results, the authors concluded that ChatGPT is promising, but that it has certain limitations in terms of use and these can be eliminated by an experienced speech-language pathologist [26]. In a study examining whether ChatGPT 3.5 can give the correct word response based on what people with aphasia say indirectly, in cases where they cannot recall words efficiently, more than 90% accuracy was achieved in the responses to examples of people with different types of aphasia [19]. These results show that ChatGPT is promising when incorporated into the therapy process. In a study designed to investigate the potential for clinical use of ChatGPT, the responses of ChatGPT to receptive and expressive language intervention activities were evaluated. As a result of this evaluation, it was found that the use of ChatGPT could provide support in creating therapy materials and working as an SLP assistant [22].

In our study, when the comprehensiveness of the responses given by ChatGPT 4.0 was evaluated it was observed that the responses given were at medium and high levels in disorders other than SSD. In SSD, the comprehensiveness of the responses was found to be at a high level in the areas of clinical decision-making and therapy planning, whereas the comprehensiveness of the responses in the task of creating assessment material and creating therapy stimuli were found to be at low levels. It is thought that comprehensiveness may have been low because ChatGPT 4.0 could not create an assessment battery and a therapy stimulation material that included all the Turkish-specific sounds in the SSD assessment material. In the tasks where the comprehensiveness parameter was concentrated at a moderate level, it was observed that the responses given by ChatGPT contained very general and superficial expressions. SLPs should pay more attention to using ChatGPT clinically in cases where ChatGPT cannot provide comprehensive responses. In a study evaluating the comprehensiveness and appropriateness of the responses provided by ChatGPT, a total of 37 questions focusing on perioperative patient education in thoracic surgery were created. Two sets of queries were sent to ChatGPT in English and Chinese for each question. The responses generated by ChatGPT were evaluated separately by experienced thoracic surgery clinicians. Both the relevance and comprehensiveness of the English and Chinese responses were high [27]. Another study evaluated the accuracy, comprehensiveness, and validity of ChatGPT compared to evidence-based sources in the diagnosis and management of common surgical conditions. Surgeons were administered a questionnaire consisting of 94 multiple-choice questions assessing the diagnostic and management knowledge generated from evidence-based sources or ChatGPT. Surgeons rated evidence-based sources as significantly more comprehensive and valid than ChatGPT, with no difference in accuracy. This suggests that while ChatGPT may offer potential benefits in practice, further refinement and validation is required to increase its usefulness and acceptance [28].

In a pre-print study evaluating the accuracy and comprehensiveness of ChatGPT in the healthcare setting, it was found that more than 50% of ChatGPT responses to 180 questions created by 33 physicians had a high level of accuracy and comprehensiveness. The same study reported that median accuracy scores were higher than mean scores in data analysis and that ChatGPT had a margin of error [29]. In line with these findings, the importance of expert-technology collaboration in the use of ChatGPT should be considered. A systematic review of the use of ChatGPT in healthcare examined studies in the literature on three levels of potential applications of ChatGPT in healthcare and/or scenarios [30]. These levels are classified as (1) general comments, (2) comments with one or more example use cases and discussion about the accuracy of their answers, and (3) in-depth discussions about the accuracy and appropriateness of their answers with qualitative and quantitative evaluation of their answers to specialised and/or scenario-specific questions [30]. There is no data on the clinical use of ChatGPT in real-world settings in the studies reviewed in this review. In that study, ChatGPT was reported to be used in areas such as clinical decision-making, preparation of information notes, counselling and research. The study also reported that ChatGPT responses were highly accurate in medical advice and education. In the studies analysed in the systematic review, ChatGPT was found to give more accurate responses as it received updates. It was observed that ChatGPT 4.0 gave better results than ChatGPT 3.5 and medical artificial intelligence models when answering questions in an examination system used to test clinical competence [31].

In our study, the relevance of ChatGPT 4.0 responses was generally found to be high. In a study examining ChatGPT responses to cancer myths, which can be considered similar to the task of creating relevant educational material in our study, it was observed that ChatGPT responses were relevant and had a high level of accuracy, similar to the responses of the National Cancer Institute [32]. In our study it was observed that the responses given by ChatGPT 4.0 to the task of creating client education material were moderately and highly accurate, comprehensive and relevant. When the relationship between the responses given by ChatGPT and the subject matter was evaluated, it was found that the performance of the responses given to the tasks of creating therapy stimuli and creating assessment materials in SSD was lower than the other tasks, while the relationship of the responses to the subject matter in other tasks and disorders was at medium and high levels. It is thought that the reason for the low performance in SSD is that Turkish-specific responses were requested. In the field of SLP, ChatGPT responses in the area of SSD, which has a low level of performance in the use of ChatGPT, should be reviewed by a speech-language pathologist. Future iterations could include high-quality datasets developed in collaboration with Turkish-speaking professionals and specifically focused on Turkish therapy resources to address this issue.

ChatGPT-4 can also be considered a low-cost solution in the field of SLP. The fact that ChatGPT-4 is available for a monthly subscription fee of $20 demonstrates its potential as a low-cost therapy assistant, especially for small clinics or SLPs. This affordable accessibility could make it possible to reach a wider audience of SLPs and therefore enable more professionals in the field of SLP to benefit from technological support.

### Limitations and future directions

While the sample size of this study is relatively small and may limit the generalizability of the findings, the study’s exploratory, descriptive design is intended to provide initial insights into the applicability of ChatGPT-4.0 in SLP tasks. Efforts were made to recruit a broader sample of SLPs with varying levels of experience; however, the length of the survey (approximately 150 min) was a barrier, leading to reluctance among potential participants. The in-depth nature of this survey was necessary to capture the nuanced evaluations of ChatGPT’s performance across diverse tasks. However, it also limited the number of individuals willing to complete the study. Despite this limitation, the data obtained from the study offer valuable insights and lay the groundwork for further research with larger, more diverse samples. Future studies with streamlined surveys or modified designs may help to overcome these challenges and increase participation, thereby enhancing the generalizability of results.

The generalizability of the study’s findings warrants careful consideration due to the predominantly academic nature of the participant sample. The sample consisted primarily of practicing SLPs who were also graduate students, which may limit the applicability of the results to broader clinical settings. While the participants’ dual roles as both clinicians and students provided valuable insights into ChatGPT’s performance from an academic and practical perspective, their evaluations may have been influenced by their exposure to academic settings and recent training. This context may not fully capture the perspectives of SLPs with extensive clinical experience but no recent academic involvement. In clinical practice, where SLPs encounter a wider range of disorders, client presentations, and situational complexities, the performance of ChatGPT may differ. For instance, SLPs in clinical practice often manage more diverse caseloads, including clients with multiple co-occurring conditions, where the ability to generate nuanced and context-specific responses becomes essential. Future research should incorporate participants from a wider range of professional backgrounds, including SLPs working exclusively in clinical environments, to better understand ChatGPT’s utility in real-world practice. Additionally, longitudinal studies could assess how ChatGPT’s effectiveness evolves as it is integrated into ongoing clinical workflows. Such studies could provide richer insights into its adaptability, the potential for learning from clinical feedback, and its impact on long-term outcomes. This approach would address the current study’s limitations and provide a more comprehensive understanding of how ChatGPT can support SLPs in diverse practice settings.

The fact that the comprehensiveness of the responses of the artificial intelligence model used in the study on linguistic and cultural diversity has yet to be fully known can be considered a limitation of the study. Therefore, the effectiveness of AI applications in different linguistic and cultural contexts should be questioned. For example, further research is needed to determine whether the responses provided by ChatGPT-4 in Turkish are as accurate and comprehensive as those provided in another language.

The effectiveness of AI in therapeutic processes has been evaluated in controlled research settings rather than in real clinical settings. Therefore, more data on performance and patient outcomes in real-world clinical applications are needed. In addition, as AI is an evolving technology, it is important to monitor its long-term effectiveness, along with updates and improvements. Additionally, future research could explore the efficiency and effectiveness of AI-generated materials compared to traditional clinician methods, specifically regarding time savings and output quality. A suggested study might involve timing participants as they perform tasks such as report writing and therapy planning using their standard templates, then comparing both the time taken and the quality of these clinician-generated outputs to those generated by ChatGPT.

It is important to address ethical concerns, risks, and practical challenges of integrating AI into clinical practice. AI systems require large amount of patient data, raising concerns about data storage, sharing, and protection, and patient privacy are critical [33]. These systems can produce misdiagnoses, especially in cases outside of training data. Also over-reliance on AI tools can reduce clinicians’ ability to think critically. High cost of developing and maintaining AI may limit accessibility for some providers. To adress these concerns, clinician training is essential, clear ethical guidelines and standards are needed to guide the use of AI in healthcare.

Only ChatGPT-4 was analysed in this study. As comparative studies with other artificial intelligence applications were not conducted, a broader perspective on the performance and effectiveness of ChatGPT-4 in the area of SLP could not be obtained. Comparing different artificial intelligence models may help to determine the most appropriate model. In addition, only text responses were received in this study. More detailed studies on the use of AI tools that generate audio, images and videos in the field of SLP are recommended. The version of ChatGPT-4.0 that we used in our study only has data up to September 2021, so this version will not have access to current information after 2021.

Future research should explore ChatGPT’s potential to improve time efficiency in SLP workflows. Time efficiency is a critical aspect of clinical practice, as SLPs are often required to balance multiple caseloads with administrative tasks. Studies could investigate how the integration of ChatGPT affects task completion times for key responsibilities such as report writing, therapy planning, and stimulus creation. For example, controlled trials could compare the time required to complete these tasks with and without the support of ChatGPT. Such investigations would provide quantitative evidence on whether ChatGPT’s use can reduce clinician workload and free up time for direct client care. Additionally, exploring the impact of ChatGPT’s time-saving potential on clinician burnout and job satisfaction would offer valuable insights into its broader implications for clinical practice.

Another promising direction for future research is the integration of multimodal AI tools alongside ChatGPT. Combining ChatGPT’s text-based capabilities with AI tools that support image, video, or audio generation could provide a more comprehensive approach to clinical support. For instance, multimodal AI systems could generate not only written therapy materials but also visual aids, instructional videos, and interactive client resources. Such tools could enhance client engagement and comprehension, particularly for individuals with communication challenges. Studies examining the synergistic effects of integrating multimodal AI tools in SLP workflows could demonstrate the feasibility, usability, and clinical outcomes of this combined approach. These future research directions could significantly contribute to advancing AI’s role in SLP and fostering broader exploration of its applications.

## Conclusions

This study evaluated ChatGPT-4’s performance across several key tasks in Speech-Language Pathology (SLP), including therapy planning, material generation, clinical decision-making, and report writing. Findings indicate that ChatGPT-4 can provide accurate, comprehensive, and relevant responses, suggesting potential time savings and support for SLPs. However, specific improvements are necessary to make AI tools like ChatGPT more effective for tasks directly related to therapy, such as therapy planning and stimulus generation. AI should incorporate more specialised datasets that reflect real-world therapeutic scenarios, including culturally diverse and language-specific materials, to better support therapy planning. Improvements in interactive feedback mechanisms, allowing AI to adapt dynamically to session progress, could also enhance its role in therapy planning. Additionally, incorporating diverse, multilingual datasets would allow AI to better handle cultural and linguistic variations, crucial for personalised therapy stimulus generation. To integrate AI into SLP effectively, SLPs would benefit from targeted training programs. These programs should focus on understanding AI’s limitations, interpreting AI-generated outputs critically, and using AI as a supportive tool rather than a primary intervention method. Furthermore, ethical considerations, such as ensuring patient data privacy and handling AI recommendations responsibly, should be core components of this training. Future research should explore these training frameworks and continue developing AI’s capabilities in culturally responsive and therapeutically relevant contexts. In certain case groups, it was observed that it gave inappropriate responses in the Turkish-specific assessment-therapy material design and therapy stimuli creation, suggesting that ChatGPT-4 needs improvement in these domains. Although an experienced speech-language pathologist can correct this situation, it shows that artificial intelligence cannot fully grasp all the details given and reveals the importance of proceeding with the guidance of an experienced speech-language pathologist in order to use such details correctly in therapy planning. The recommendations of ChatGPT-4 may be promising as a starting point for decisions based on expertise and clinical experience, but it should be under expert supervision for final decisions.

## Electronic supplementary material

Below is the link to the electronic supplementary material.

Supplementary Material 1

## Data Availability

The data that support the findings of this study are available from the corresponding author upon reasonable request.
