# Correction: Is there any room for ChatGPT AI bot in speech-language pathology?

**DOI:** 10.1007/s00405-025-09363-3

**Published:** 2025-05-08

**Authors:** Namık Yücel Birol, Hilal Berber Çiftci, Ayşegül Yılmaz, Ayhan Çağlayan, Ferhat Alkan

**Affiliations:** 1 Department of Speech and Language Therapy, Faculty of Health Sciences, Tarsus University, Mersin, Türkiye; 2 Department of Speech and Language Therapy, Graduate School of Health Sciences, İstanbul Medipol University, İstanbul, Türkiye; 3 Çağlayan Speech and Language Therapy Center, İzmir, Türkiye; 4 Department of Speech and Language Therapy, Institute of Graduate Education, İstinye University, İstanbul, Türkiye

**European Archives of Oto-Rhino-Laryngology**

10.1007/s00405-025-09295-y

In this article, the sentence in the sub-section “Procedure” of the method section was incorrectly published as ‘Approval was obtained from the Non-Interventional Clinical Ethics Committee of XXX University on XXX date, with the decision of the ethics committee numbered XXX, stating that there was no ethical problem in conducting this research’ but should read as ‘Approval was obtained from the Non-Interventional Clinical Ethics Committee of Cappadocia University on April 14, 2023, with the decision of the ethics committee numbered E-64577500-050.99-44211, stating that there was no ethical problem in conducting this research’.

The original article has been corrected.

