# Supplementary material for: Is there any room for ChatGPT AI bot in speech-language pathology?
Source: Eur Arch Otorhinolaryngol. 2025 Mar 1;282(6):3267–80. doi: 10.1007/s00405-025-09295-y (PMC12122639; doi:10.1007/s00405-025-09295-y)
Supplement: Supplementary file 1 — Supplementary Material 1 [file 405_2025_9295_MOESM1_ESM.docx]

**Supplementary Information – ChatGPT Prompts and Responses**

**1a) Speech Sound Disorders - Report Writing**

| **Prompt:**  Write a speech and language assessment report according to the information I will give you below.  The client's name is Ahmet Yılmaz. Ahmet is 7 years and 5 months old. Speech and language assessment was carried out at Türkiye University Speech and Language Therapy Unit on 19 April 2023. Ahmet was assessed by the speech and language therapist Fatma Uslu. Ahmet's mother stated that Ahmet produced some sounds incorrectly. Ahmet's consonant production at the word level was evaluated with the Articulation Subtest of the Turkish Articulation and Phonology Test (SST). Ahmet lateralized /s/ and /z/ in all positions. Ahmet is stimulable for /s/ and /z/ at the word level. Ahmet's orofacial mechanism is structurally and functionally normal. Ahmet's results on diadochokinetic syllable tasks are consistent with typically developing peers. Hearing screening was performed at 20 dB for frequencies of 250, 500, 1000, 2000, 4000 and 6000 Hz. Ahmet responded bilaterally to all sounds. Turkish Early Language Development Test (TEDIL) was applied to evaluate Ahmet's language skills. The results of TEDIL were compatible with his typically developing peers in receptive and expressive language skills. Ahmet's voice and fluency skills are normal. |
| --- |
| **ChatGPT Answer:**  **SPEECH AND LANGUAGE ASSESSMENT REPORT**  **Client Information:**  - Client Name: Ahmet Yılmaz  - Age: 7 years and 5 months  - Date of Assessment: 19 April 2023  - Location: Türkiye University Speech and Language Therapy Unit  - Assessor: Fatma Uslu, Speech and Language Therapist  **Referral Information:**  Ahmet's assessment was prompted by his mother's concern regarding his incorrect production of certain sounds.  **Assessment Procedures and Results:**  *Articulation Skills*  The Articulation Subtest of the Turkish Articulation and Phonology Test (SST) was administered to assess Ahmet's consonant production at the word level. Ahmet showed difficulty in articulating /s/ and /z/, demonstrating lateralization in all positions. However, it is important to note that he is stimulable for /s/ and /z/ at the word level.  *Oral Motor Examination*  An examination of Ahmet's orofacial mechanism showed no structural or functional abnormalities. His orofacial mechanism is functioning at a level commensurate with his typically developing peers.  *Diadochokinetic Syllable Task*  Ahmet's performance on the diadochokinetic syllable tasks is consistent with the results of typically developing peers.  *Hearing Screening*  A hearing screening was performed at 20 dB for frequencies of 250, 500, 1000, 2000, 4000, and 6000 Hz. Ahmet responded appropriately to all sounds bilaterally, indicating normal auditory processing.  *Language Skills*  The Turkish Early Language Development Test (TEDIL) was used to evaluate Ahmet's receptive and expressive language skills. Ahmet's results fell within the typical range when compared to his peers.  *Voice and Fluency Skills*  Upon evaluation, Ahmet's voice and fluency skills were found to be within normal limits.  **Summary:**  While Ahmet demonstrates lateralization in the pronunciation of the /s/ and /z/ sounds in all positions, he also exhibits stimulability for these sounds at the word level. This issue seems to be the sole area of concern as the remaining aspects of his speech and language skills - including his orofacial mechanism, diadochokinetic syllable tasks, hearing, language skills, and voice and fluency skills - are consistent with typically developing peers.  **Recommendations:**  Considering Ahmet's stimulability for correct /s/ and /z/ production, a course of speech therapy focused on the correct articulation of these sounds is recommended. Regular monitoring and review of his progress would be beneficial to ensure continued development of his speech and language skills. |

**1b) Speech Sound Disorders - Creating Assessment Material**

| **Prompt:**  Create sentences containing each consonant in Turkish in all syllable positions to assess speech sound production. |
| --- |
| **ChatGPT Answer:**  In Turkish, there are 21 consonants: b, c, ç, d, f, g, ğ, h, j, k, l, m, n, p, r, s, ş, t, v, y, z. Let's create sentences that include each of these consonants in the initial, medial, and final position of a syllable.  Initial position:  1. Bize bak, bütün bayrakları bir arada görüyoruz.  2. Cem cenini caddede yürüyerek çaldı.  3. Çocuk çayı çalkalayarak içti.  4. Dün dersi dikkatle dinledim.  5. Fatma, ferah bir fikir fırlattı.  6. Gül, gözlüklerini güvenli bir yere götürdü.  7. Ğaileli ğuldu, ğildan yana döndü.  8. Hüseyin, hızla haneye gitti.  9. Jale, jetonu jakarandaların yanına attı.  10. Kız kardeşiyle kafede buluştu.  11. Lalelerle dolu bir bahçe gördüm.  12. Mehmet, mağarasını muhteşem bir yerde kurdu.  13. Nur, nalburdan naylon bir çanta aldı.  14. Pazarı panayırda kurduk.  15. Recep, rüzgarlı bir günde rahatlamaya gitti.  16. Selin, sinemada sakin bir film seyretti.  17. Şirin, şalvarını şık bir şekilde giydi.  18. Teyzem, torunuyla teneffüse çıktı.  19. Veli, vadide vahşi bir hayvan gördü.  20. Yeni yıl yemeklerini yeşil bir alanda yaptık.  21. Zeynep, zembereklerini zarif bir kutuda sakladı.    Medial position:  1. Sabahtan akşama kadar balık avladık.  2. İçeceğimiz suyu açık bir çeşmeden aldık.  3. Hocanın dersleri oldukça bilgilendiriciydi.  4. Safranbolu'dan sıra dışı bir hediye aldım.  5. Gezgin, bütün gün ormanda kamp kurdu.  6. Karabağ'da elma yetiştirmeye başladık.  7. Kitapçı, kahve içerken öğle uykusuna daldı.  8. Yarışmacılar, yüzlerini yıkadıktan sonra kahvaltıya oturdular.  9. Japonca öğrenmek için kitap okuyorum.  10. Gökten kar yağdığını fark ettim.  11. Öğle yemeği için balıklı pilav yaptık.  12. Annesi çocuğun yanına oturdu.  13. Küçük papağan, tünekten aşağıya atladı.  14. Şarap kadehini dikkatli bir şekilde yerleştirdi.  15. Ressam, eserini sergileyerek halkın beğenisine sundu.  16. Şehrin sokaklarını gezerken bir kitapçıya rastladım.  17. Kapıdan çıktığımda yağmur yağmaya başladı.  18. Üniversitede tanıştığımız kişi çok ilginç biriydi.  19. Van'dan çıkan vapur Bodrum'a doğru ilerledi.  20. Seyyah, yaylalarda gezerken ilginç bir kelebek gördü.  21. Müzik, her zaman zihnimizi rahatlatır.    Final position:  1. Bu kitabı bitirdim.  2. Çorabı çamaşır sepetine attım.  3. Dersin sonunda hoca bir test uyguladı.  4. Farklı bir fikir düşünmeliyiz.  5. Görüşmek üzere dedi ve gitti.  6. Ğ harfini doğru telaffuz etmek zordur.  7. Üşüdüm, bir hırka giyeceğim.  8. Bahçedeki ağaçta bir jilet buldum.  9. Sütten yoğurt yaptık.  10. Çocukları okula gönderdik.  11. İlk buluşmamızı asla unutamam.  12. Anılarına sıkı sıkıya bağlıydı.  13. Pınardan su içti.  14. Kitabın kapak resmini çok beğendim.  15. Evin bahçesinde bir sera yapmayı düşünüyoruz.  16. Şehirdeki yeni parkta biraz yürüdük.  17. Öğretmenin tavsiyelerini ciddiye almalıyız.  18. Çocuklar parkta voleybol oynadılar.  19. Şehirdeki her ev beyazdı.  20. Kıyıda bir yengeç buldum.  21. Her zaman doğruyu söylemelisin. |

**1c) Speech Sound Disorders - Clinical Decision Support**

| **Prompt:**  Support me in making a clinical decision about the client whose details I have given below.  The client's name is Ahmet Yılmaz. Ahmet is 7 years and 5 months old. Speech and language assessment was carried out at Türkiye University Speech and Language Therapy Unit on 19 April 2023. Ahmet was assessed by the speech and language therapist Fatma Uslu. Ahmet's mother stated that Ahmet produced some sounds incorrectly. Ahmet's consonant production at the word level was evaluated with the Articulation Subtest of the Turkish Articulation and Phonology Test (SST). Ahmet lateralized /s/ and /z/ in all positions. Ahmet is stimulable for /s/ and /z/ at the word level. Ahmet's orofacial mechanism is structurally and functionally normal. Ahmet's results on diadochokinetic syllable tasks are consistent with typically developing peers. Hearing screening was performed at 20 dB for frequencies of 250, 500, 1000, 2000, 4000 and 6000 Hz. Ahmet responded bilaterally to all sounds. Turkish Early Language Development Test (TEDIL) was applied to evaluate Ahmet's language skills. The results of TEDIL were compatible with his typically developing peers in receptive and expressive language skills. Ahmet's voice and fluency skills are normal. |
| --- |
| **ChatGPT Answer:**  Based on the information you've provided, Ahmet seems to have an articulation disorder specifically affecting his /s/ and /z/ sounds, as he's lateralizing these sounds in all positions. The rest of his language, voice, fluency skills, and his oral mechanism seem to be within the normal range for his age, as per the results of the Turkish Articulation and Phonology Test (SST), Turkish Early Language Development Test (TEDIL), diadochokinetic syllable tasks, and hearing screening.  Here are my recommendations for a clinical decision:  **1. Articulation Therapy:** Given Ahmet's stimulability for /s/ and /z/ sounds at the word level, individualized speech therapy focusing on these sounds would be beneficial. Techniques such as auditory discrimination, phonetic placement, and drill practice might be helpful in therapy.  **2. Regular Monitoring:** Although Ahmet's language development seems to be on track currently, it is recommended to continue monitoring his progress regularly to ensure there are no further issues in the future.  **3. Home Practice:** Ahmet's parents can be guided to help him practice the correct articulation at home. The speech therapist can provide them with instructions and exercises to work on outside of therapy sessions.  **4. Follow-up Assessment:** Schedule a follow-up assessment after a few months of therapy to evaluate progress and adjust the treatment plan if necessary.  Please note that this advice should be considered alongside clinical judgement and any other relevant information about Ahmet's medical history and personal circumstances. It's also essential to communicate clearly with Ahmet and his parents throughout this process to ensure they understand the therapy goals and processes. |

**1d) Speech Sound Disorders - Creating Therapy Stimuli**

| **Prompt:**  Find meaningful Turkish p-b minimal pairs for voicing phonological process. |
| --- |
| **ChatGPT Answer:**  A minimal pair is a pair of words that differ by only a single sound in the same position and have different meanings. In the context of phonetics, a "p-b" minimal pair would refer to a pair of words where the only difference is the use of "p" in one word and "b" in the other.    Since Turkish has both /p/ and /b/ phonemes and the phonemic inventory of Turkish doesn't include voiced and voiceless pairs at the end of the words, the examples should be from the beginning or the middle of the words. Here are some examples of "p-b" minimal pairs in Turkish:  1. "pala" (saber) vs. "bala" (honeychild)  2. "pil" (battery) vs. "bil" (know)  3. "pazar" (market/Sunday) vs. "bazar" (bazaar)  4. "pati" (paw) vs. "bati" (west)  5. "kapı" (door) vs. "kabı" (its container)  Remember that the comparison is made according to the pronunciation, not the spelling, since in Turkish spelling and pronunciation are directly correlated. Also, remember to check the meaning of words in the context they are used, as some Turkish words can have different meanings. |

**1e) Speech Sound Disorders - Therapy Planning**

| **Prompt:**  Create a speech therapy plan for the client with a articulation disorder, whose information I have given below.  The client's name is Ahmet Yılmaz. Ahmet is 7 years and 5 months old. Speech and language assessment was carried out at Türkiye University Speech and Language Therapy Unit on 19 April 2023. Ahmet was assessed by the speech and language therapist Fatma Uslu. Ahmet's mother stated that Ahmet produced some sounds incorrectly. Ahmet's consonant production at the word level was evaluated with the Articulation Subtest of the Turkish Articulation and Phonology Test (SST). Ahmet lateralized /s/ and /z/ in all positions. Ahmet is stimulable for /s/ and /z/ at the word level. Ahmet's orofacial mechanism is structurally and functionally normal. Ahmet's results on diadochokinetic syllable tasks are consistent with typically developing peers. Hearing screening was performed at 20 dB for frequencies of 250, 500, 1000, 2000, 4000 and 6000 Hz. Ahmet responded bilaterally to all sounds. Turkish Early Language Development Test (TEDIL) was applied to evaluate Ahmet's language skills. The results of TEDIL were compatible with his typically developing peers in receptive and expressive language skills. Ahmet's voice and fluency skills are normal. |
| --- |
| **ChatGPT Answer:**  Client: Ahmet Yılmaz  Age: 7 years and 5 months  Disorder: Articulation Disorder - lateralized /s/ and /z/  Assessment Team: Fatma Uslu, Speech and Language Therapist, Türkiye University Speech and Language Therapy Unit  Speech Therapy Plan:  The main goal of this speech therapy plan is to correct the articulation of /s/ and /z/ sounds. Given that Ahmet is stimulable for /s/ and /z/ at the word level, we will focus on practicing these sounds to increase accuracy in all positions. The therapy plan will be carried out over 12 weeks, with weekly sessions of 45 minutes each.  Week 1 - Week 3:  • Objective: Increase awareness of correct /s/ and /z/ production.  • Activity: The therapist will model correct production of /s/ and /z/ sounds at the word level. Ahmet will be asked to listen and compare correct and incorrect sounds. Ahmet will also practice producing /s/ and /z/ sounds in isolation, guided by the therapist.  Week 4 - Week 6:  • Objective: Improve correct articulation of /s/ and /z/ sounds at the word level.  • Activity: Ahmet will practice articulating /s/ and /z/ sounds at the beginning, middle, and end of words. The therapist will provide immediate feedback and correction. We will use age-appropriate words that Ahmet frequently uses in his speech.    Week 7 - Week 9:  • Objective: Transfer correct articulation of /s/ and /z/ sounds to sentence level.  • Activity: Ahmet will be given sentences that include words with /s/ and /z/ sounds. He will practice reading these sentences out loud and participating in guided conversation.  Week 10 - Week 12:  • Objective: Promote generalization of correct /s/ and /z/ articulation in conversational speech.  • Activity: Ahmet will engage in structured and unstructured conversation with the therapist, peers, and family members. The therapist will provide minimal corrections to promote confidence and fluency.  Home Practice:  Ahmet's parents will be given instructions and materials for home practice. They will be encouraged to engage Ahmet in activities that provide opportunities to practice /s/ and /z/ sounds. Home practice should be carried out for about 15 minutes each day.  Continuous Assessment:  Progress will be monitored continuously throughout the therapy period. Adjustments to the plan will be made as necessary based on Ahmet's progress.  Discharge Plan:  If Ahmet can consistently produce the /s/ and /z/ sounds correctly in conversational speech across different settings (therapy, home, school), he will be discharged from therapy. A follow-up assessment will be scheduled 3 months post-therapy to ensure maintenance of skills.  This plan is meant to be flexible and adaptable to Ahmet's needs and progress. His motivation, comfort, and emotional well-being will be a priority throughout the process. The involvement and cooperation of his parents will be crucial for successful home practice and generalization of learned skills. |

**1f) Speech Sound Disorders - Creating Education Material**

| **Prompt:**  Create a client education material for childhood apraxia of speech. |
| --- |
| **ChatGPT Answer:**  **Understanding Childhood Apraxia of Speech: An Educational Guide for Parents**  **1. What is Childhood Apraxia of Speech (CAS)?**  Childhood Apraxia of Speech (CAS) is a motor speech disorder that makes it hard for children to speak. It is not due to muscle weakness or paralysis, but rather the brain struggles to develop plans for speech movement. As a result, your child might find it hard to accurately produce speech sounds and sequences.  **2. How is CAS Diagnosed?**  Diagnosis is typically made by a speech-language pathologist (SLP) through a comprehensive evaluation of your child’s speech and language, oral-motor abilities, and vocal quality. Other conditions may be ruled out first, as CAS has shared symptoms with various other speech and language disorders.  **3. Common Signs of CAS**  Children with CAS often:  - Have inconsistent speech errors.  - Can understand language better than they can express it.  - May appear to be groping when attempting to produce sounds or coordinate the lips, tongue, and jaw for purposeful movement.  - Have more difficulty saying longer or more complex words.  - Show slow progress in speech therapy.  **4. Causes of CAS**  The cause of CAS is often unknown, but it may be associated with brain damage, genetic disorders, or neurological conditions. However, many children with CAS have no known neurological condition or injury. CAS is a complex disorder, and research is ongoing to better understand its causes.  **5. Treatment for CAS**  CAS is treated with speech therapy, usually provided by a speech-language pathologist. The specific techniques and goals can vary depending on the child's age, symptoms, and developmental stage. Therapy often includes:  - Extensive work on speech drills to improve the child's ability to produce certain sounds and sound sequences.  - Using other forms of communication, like sign language or picture boards, while the child works on speech skills.  - Frequent and intensive therapy sessions, sometimes up to 3-5 times per week.  **6. Supporting Your Child at Home**  Here are some ways you can help your child:  - Repeat what your child says, providing a model for correct pronunciation.  - Sing songs and do rhymes to practice speech rhythm and inflection.  - Use picture books to work on naming and describing items.  - Be patient and provide plenty of opportunities for your child to communicate in low-stress situations.  **7. What to Expect in the Future?**  Every child with CAS is unique. With early intervention and appropriate treatment, many children can improve their speech significantly. The process is slow and requires consistent effort, but progress is possible.  **8. Helpful Resources**  - Apraxia Kids (www.apraxia-kids.org)  - American Speech-Language-Hearing Association (www.asha.org)  - National Institute on Deafness and Other Communication Disorders (www.nidcd.nih.gov)  Remember, you are not alone in this journey. There is support available, both from professionals and from other parents of children with CAS. Keep advocating for your child, and together, you can overcome the challenges that CAS presents. |

**2a) Motor Speech Disorders -  Report Writing**

| **Prompt:**  Write a speech assessment report according to the information I will give you below.  The client's name is Ayşe Gedik. Ayşe is 60 years old. She presented with a three-year history of progressive stiffness during the walk, difficulty turning in bed and handwriting. She also presented two-year history of speech difficulty. On neurologic examination, her arm swings were diminished and her extremities were rigid. She had moderate static tremors in both hands. She had generalized bradykinesia. Facial expression was masked. The MRI scan was normal. Speech assessment was carried out at Türkiye University Speech and Language Therapy Unit on 19 April 2023. Ayşe was assessed by the speech and language therapist Fatma Uslu. The oral mechanism exam revealed the reduced range of movement on non-speech tasks; lip and jaw static tremors. The speech examination on conversation and reading revealed reduced loudness; imprecise articulation; accelerated rate; monopitch, monoloudness and reduced range of articulatory movement. Speech alternating motion rates were fast and blurred. Prolonged “ah” was breathy-hoarse voice quality. Resonance was normal. The neurologist concluded that the patient had idiopathic Parkinson's disease and the speech-language pathologist diagnosed the patient had hypokinetic dysarthria. |
| --- |
| **ChatGPT Answer:**  **Speech Assessment Report**  **Client Details:**  Name: Ayşe Gedik  Age: 60 Years  Date of Assessment: 19th April 2023  Location: Türkiye University Speech and Language Therapy Unit  Speech and Language Therapist: Fatma Uslu  **Referral Information and Background:**  Mrs. Gedik presented with a three-year history of increasing stiffness during walks, difficulty turning in bed, and declining handwriting quality. Additionally, she exhibited a two-year history of speech difficulty. Neurologic examination noted diminished arm swings, rigidity in extremities, moderate static tremors in both hands, generalized bradykinesia, and masked facial expression. An MRI scan did not reveal any anomalies.  **Oral Mechanism Exam Findings:**  The assessment of the oral mechanism revealed a reduced range of movement during non-speech tasks, along with observable lip and jaw static tremors.  **Speech Assessment Findings:**  In conversational and reading contexts, Mrs. Gedik showed reduced loudness, imprecise articulation, and an accelerated rate of speech. Her vocal output was marked by monopitch and monoloudness, suggesting a diminished capacity for dynamic vocal expression. Furthermore, a reduced range of articulatory movement was noted. Speech alternating motion rates were found to be fast and blurred. Vocalization of a prolonged "ah" exhibited a breathy-hoarse voice quality, however, resonance remained within the normal range.  **Diagnosis:**  In light of Mrs. Gedik's overall clinical picture, her neurologist determined the root cause to be idiopathic Parkinson's disease. Correspondingly, as a result of the speech and language assessment, Mrs. Gedik has been diagnosed with hypokinetic dysarthria by the speech-language pathologist. This speech disorder, typically associated with Parkinson's disease, aligns with the aforementioned symptoms of reduced vocal volume, articulation imprecision, rapid speech rate, monotonous pitch and volume, and a breathy-hoarse voice quality.  **Recommendations:**  It is recommended that Mrs. Gedik engage in regular speech and language therapy sessions to manage her hypokinetic dysarthria symptoms. The treatment plan should focus on slowing the rate of speech, improving articulation and vocal volume, as well as addressing voice quality issues.  As the stiffness and tremors characteristic of Parkinson's disease could make therapy challenging, it is also suggested that her care team liaise closely with her neurologist to monitor her condition and ensure that her treatment regimen complements her overall management plan. |

**2b) Motor Speech Disorders -  Creating Assessment Material**

| **Prompt:**  Create a Dysarthria Assessment Battery for assessing dysarthria. |
| --- |
| **ChatGPT Answer:**  Sure, I can help you outline a basic Dysarthria Assessment Battery. Dysarthria is a motor speech disorder resulting from neurological injury, and it affects the physical production of speech. Patients may present with slurred or slow speech, limited tongue, lip, and jaw movement, or abnormal pitch and rhythm.  The Dysarthria Assessment Battery may include the following components:  **1. Medical and Case History**     - Understanding the patient's medical history, onset, and progression of symptoms.     - Establishing the patient's functional communication status (e.g., conversation, use of phone, etc.).     - Evaluating the patient's perceived impairments and their impact on daily living.  **2. Oral Motor Examination**     - Assessing the structure and function of the speech mechanism (lips, tongue, jaw, velum).     - Evaluating the strength, range of motion, and accuracy of movement for speech and non-speech tasks.  **3. Perceptual Speech Assessment**     - Analyzing articulation, phonation, resonance, prosody, and rate of speech.     - Understanding breath support for speech and voice.     - Use of standardized assessment tools like the Frenchay Dysarthria Assessment (FDA-2), Dysarthria Profile (DP), or Assessment of Intelligibility of Dysarthric Speech (AIDS).  **4. Acoustic Analysis**     - Measuring pitch, loudness, duration, voice quality, and variability using spectrographic analysis.     - Analyzing speech timing (pauses, rate, rhythm, stress).  **5. Aerodynamic Assessment**     - Evaluating respiratory, phonatory, and articulatory systems to assess air pressure, airflow, and voice onset time.  **6. Dysphagia Assessment**     - Since dysarthria and dysphagia often coexist, it is essential to evaluate swallowing function.  **7. Functional Communication Assessment**     - Assessing how well the person is able to communicate in daily life situations.     - Measuring the impact of dysarthria on the person’s quality of life using tools such as the Dysarthria Impact Profile.  **8. Cognitive-Communication Assessment**     - For some types of dysarthria, especially those related to progressive neurological conditions, assessing cognitive-communication abilities is also important.  Remember, this is just a guideline and actual assessments may need to be tailored to meet the specific needs and abilities of the individual patient. And of course, a licensed speech-language pathologist should perform the assessment and interpret the results.  After the assessment, an appropriate therapy plan can be devised, which may include speech therapy, use of augmentative and alternative communication (AAC), or referral to other specialists as needed. |

**2c) Motor Speech Disorders - Clinical Decision Support**

| **Prompt:**  Support me in making a clinical decision about the clients' differential speech diagnosis, whose details I have given below.  The client's name is Ayşe Gedik. Ayşe is 60 years old. She presented with a three-year history of progressive stiffness during the walk, difficulty turning in bed and handwriting. She also presented two-year history of speech difficulty. On neurologic examination, her arm swings were diminished and her extremities were rigid. She had moderate static tremors in both hands. She had generalized bradykinesia. Facial expression was masked. The MRI scan was normal. Speech assessment was carried out at Türkiye University Speech and Language Therapy Unit on 19 April 2023. Ayşe was assessed by the speech and language therapist Fatma Uslu. The oral mechanism exam revealed the reduced range of movement on non-speech tasks; lip and jaw static tremors. The speech examination on conversation and reading revealed reduced loudness; imprecise articulation; accelerated rate; monopitch, monoloudness and reduced range of articulatory movement. Speech alternating motion rates were fast and blurred. Prolonged “ah” was breathy-hoarse voice quality. Resonance was normal. The neurologist concluded that the patient had idiopathic Parkinson's disease. |
| --- |
| **ChatGPT Answer:**  Based on the information provided, it appears that Ayşe Gedik is experiencing hypokinetic dysarthria, a type of speech disorder often associated with Parkinson's disease. Let's discuss the key findings supporting this differential speech diagnosis.  **1. Reduced range of movement on non-speech tasks; lip and jaw static tremors:** Hypokinetic dysarthria often involves reduced movements of the articulators, including the lips, jaw, and tongue, which contributes to the imprecise articulation observed.  **2. Reduced loudness; imprecise articulation; accelerated rate; monopitch, monoloudness, and reduced range of articulatory movement:** These features are characteristic of hypokinetic dysarthria. Reduced loudness and rapid, blurred speech are particularly indicative of this condition.  **3. Fast and blurred speech alternating motion rates (AMRs):** Individuals with hypokinetic dysarthria often exhibit accelerated AMRs, leading to blurred speech as seen in this case.  **4. Breathy-hoarse voice quality during the prolonged "ah" task:** While Parkinson's patients often have a monotonous, low-volume voice, some can present with a breathy or hoarse voice due to insufficient closure of the vocal folds. This could be due to rigidity and bradykinesia affecting laryngeal muscles.  **5. Normal resonance:** This is expected in hypokinetic dysarthria, as resonance disorders are not typically associated with Parkinson's disease.    The normal MRI scan and the symptoms of generalized bradykinesia, facial masking, and static tremors in hands reported by the neurologist confirm the diagnosis of Parkinson's disease, which is a common cause of hypokinetic dysarthria.  For managing Ayşe's condition, Speech and Language Therapy should focus on enhancing the intelligibility and volume of her speech. Lee Silverman Voice Treatment (LSVT LOUD®), for example, is a speech treatment approach specifically designed for people with Parkinson's. It focuses on improving vocal loudness, which can enhance articulatory precision, voice quality, and even facial expression. This treatment approach might be beneficial for Ayşe, and I would recommend discussing this option with her and her family.  However, always remember that while speech therapy can provide significant improvements, it should be part of a larger, interdisciplinary approach to managing Ayşe's Parkinson's disease, which may include medication, physical therapy, occupational therapy, and potentially other interventions. Coordination with her neurologist and other healthcare providers will be crucial for ensuring Ayşe receives comprehensive care. |

**2d) Motor Speech Disorders - Creating Therapy Stimuli**

| **Prompt:**  Create 10 functional phrases for a client with hypokinetic dysarthria to use in Lee Silverman Voice Treatment (LSVT LOUD®). |
| --- |
| **ChatGPT Answer:**  Lee Silverman Voice Treatment (LSVT LOUD®) is a type of speech therapy designed specifically for individuals with Parkinson's disease and other neurological conditions that cause hypokinetic dysarthria. This type of therapy focuses on increasing vocal loudness and improving articulation through a series of exercises and functional phrases. Here are ten functional phrases that can be used during LSVT LOUD® therapy.  1. "Good morning, how are you today?"  2. "I would like a cup of coffee, please."  3. "Can we go for a walk in the park?"  4. "What is the weather forecast for tomorrow?"  5. "Let's meet for lunch at our favorite place."  6. "I need to make an appointment with my doctor."  7. "Can you help me find my glasses?"  8. "Where is the nearest grocery store?"  9. "I love spending time with my grandchildren."  10. "Please remember to lock the front door."  These phrases are everyday sentences that involve a range of phonetic content and require various movements of the speech apparatus. By repeating these phrases in an excessively loud and clear manner, individuals with hypokinetic dysarthria can improve their articulatory precision, vocal intensity, and overall speech intelligibility. The phrases are also designed to be useful in daily life, reinforcing the practical application of the exercises. Remember, consistent practice is key to improving speech skills in individuals with hypokinetic dysarthria. |

**2e) Motor Speech Disorders - Therapy Planning**

| **Prompt:**  Create a speech therapy plan for the client with a hypokinetic dysarthria, whose information I have given below.  The client's name is Ayşe Gedik. Ayşe is 60 years old. She presented with a three-year history of progressive stiffness during the walk, difficulty turning in bed and handwriting. She also presented two-year history of speech difficulty. On neurologic examination, her arm swings were diminished and her extremities were rigid. She had moderate static tremors in both hands. She had generalized bradykinesia. Facial expression was masked. The MRI scan was normal. Speech assessment was carried out at Türkiye University Speech and Language Therapy Unit on 19 April 2023. Ayşe was assessed by the speech and language therapist Fatma Uslu. The oral mechanism exam revealed the reduced range of movement on non-speech tasks; lip and jaw static tremors. The speech examination on conversation and reading revealed reduced loudness; imprecise articulation; accelerated rate; monopitch, monoloudness and reduced range of articulatory movement. Speech alternating motion rates were fast and blurred. Prolonged “ah” was breathy-hoarse voice quality. Resonance was normal. The neurologist concluded that the patient had idiopathic Parkinson's disease. |
| --- |
| **ChatGPT Answer:**  Speech Therapy Plan for Ayşe Gedik  Client: Ayşe Gedik  Age: 60 years old  Diagnosis: Idiopathic Parkinson's Disease with Hypokinetic Dysarthria  Therapist: Fatma Uslu  Date: 10th July 2023  I. Goals of Therapy:  1. To improve the speech intelligibility by working on articulation and phonation.  2. To increase the vocal loudness and control rate of speech.  3. To improve the orofacial muscle strength and movement for both speech and non-speech tasks.  4. To enhance the communication skills and overall quality of life.  II. Treatment Strategies:  1. Lee Silverman Voice Treatment (LSVT LOUD): This is a well-established program specially designed for people with Parkinson’s Disease to improve their vocal loudness, intonation, and voice quality.  2. Articulation Therapy: The aim will be to improve the precision of speech sounds, which will in turn improve the intelligibility of Ayşe's speech. Exercises will be given to improve tongue, lip, and jaw movements.  3. Rate Control: Techniques will be used to slow down Ayşe's speech rate. This can be accomplished through pacing strategies, like using a metronome or visual cues.  4. Non-Speech Orofacial Exercises: These exercises are aimed at improving the range of movement and reducing rigidity in Ayşe's face and jaw.  III. Session Plan:  1. Warm-up exercises including deep breathing and orofacial muscle stretching.  2. LSVT LOUD exercises focusing on long 'ah' sounds and maintaining a consistent loud voice.  3. Articulation exercises focusing on sounds that Ayşe finds most challenging.  4. Rate control exercises using pacing strategies.  5. Non-speech orofacial exercises to improve range of movement and reduce facial rigidity.  6. Cool down exercises and vocal hygiene education.  IV. Frequency of Sessions:  Ideally, therapy sessions should be conducted four times a week for a duration of four weeks as per the standard LSVT LOUD protocol. However, the frequency and duration of the therapy may be adjusted according to Ayşe's progress and tolerance.  V. Progress Monitoring:  Ayşe's progress will be monitored throughout the therapy using objective measures like voice recordings and subjective measures like self-reports about improvements in daily communication. Regular meetings with Ayşe and her family members will be held to discuss progress and adjust treatment strategies if needed.  In conclusion, the proposed therapy plan aims to address the challenges faced by Ayşe due to her hypokinetic dysarthria caused by Parkinson's Disease. By focusing on the key areas of articulation, voice quality, rate of speech, and orofacial movements, the plan aims to improve Ayşe's speech intelligibility and her overall quality of life. |

**2f) Motor Speech Disorders - Creating Education Material**

| **Prompt:**  Create a client education material for hypokinetic dysarthria. |
| --- |
| **ChatGPT Answer:**  **Client Education Material: Understanding Hypokinetic Dysarthria**  **What is Hypokinetic Dysarthria?**  Hypokinetic dysarthria is a motor speech disorder characterized by difficulties in the muscles that produce speech. It is caused by damage to the basal ganglia control circuit in the brain, typically associated with Parkinson’s disease.  **Signs and Symptoms**  While each individual’s experience with hypokinetic dysarthria may differ, common signs and symptoms include:  - Reduced volume and monotone voice.  - Rapid, mumbled, or slurred speech.  - Difficulty starting or stopping speech.  - Short rushes of speech.  - Difficulty articulating certain sounds or words.  **How Is Hypokinetic Dysarthria Diagnosed?**  The diagnosis of hypokinetic dysarthria typically involves a multi-step process:  **1. Medical History:** A thorough review of your medical history helps determine whether there has been any damage to your nervous system.  **2. Physical Examination:** An examination of your speech and voice, including how clearly you speak and the quality of your voice.  **3. Speech-Language Pathologist Assessment:** An in-depth evaluation of your speech abilities by a trained speech-language pathologist.  **Treatment and Management**  While there is currently no cure for hypokinetic dysarthria, treatment focuses on managing symptoms and improving communication abilities. Your specific treatment plan will be designed by your speech-language pathologist and may include:  **- Speech Therapy:** This can help improve your articulation, breathing, and voice quality. Therapy may include exercises to increase tongue, lip, and jaw flexibility and strength, as well as techniques to slow down your speech rate.  **- Assistive Technology:** In some cases, using speech-generating devices or augmentative and alternative communication (AAC) aids can help support communication.  **- Medication and/or Surgery:** These may be recommended by your doctor to address underlying neurological conditions, like Parkinson’s disease, that may be contributing to your speech difficulties.  **Living with Hypokinetic Dysarthria**  Living with hypokinetic dysarthria can be challenging, but there are several strategies to make communication easier:  **1. Speak Slowly:** Try to slow down your speech. This can help make your words more clear and easy to understand.  **2. Pause Regularly:** Pausing between words or phrases can help others understand you more easily.  **3. Use Short Sentences:** Short, simple sentences are often easier for others to understand.  **4. Face-to-Face Communication:** Face-to-face conversations can be easier as it allows the listener to use visual cues.  Remember, everyone's journey with hypokinetic dysarthria is unique. It is okay to ask for help, and there are many resources available to you. Your speech-language pathologist is there to support you and work with you on improving your communication skills. It's important to be patient with yourself and understand that progress may be slow, but every step forward counts.  *Note: This educational material is intended to provide a basic understanding of hypokinetic dysarthria. It does not replace the advice or treatment recommendations of a healthcare professional. Always consult with your healthcare provider for a diagnosis and treatment plan.* |

**3a) Aphasia -  Report Writing**

| **Prompt:**  Write a speech and language assessment report according to information I will give you below  The client's name is Emre Kara. He is a 60 years old Turkish patient who had a stroke in the left hemisphere middle cerebral artery of his brain due to hypertension three months ago. His vision and hearing is normal. There is a history of stay in intensive care unit for two days. He was hospitalized for 15 days. He was evaluated by Fatma Uslu, Speech and Language Therapist at the Speech and Language Therapy Unit of Türkiye University. He is married and he has one child. He was a teacher and he is retired. He has right sided hemiparesis and he has aphasia. His cooperation is good. His mini mental test score is 26. He was evaluated for motor speech disorder. He has not dysartria or apraxia of speech. His swallowing is normal. Turkish Aphasia Language Assessment Test (TALAT) was performed and according to test his scores was 12 points for speaking fluency, 50 points for auditory comprehension, 8 points for repetition, 14 points for naming, 20 points for reading, 8 points for grammar, 8 points for speech actions, and 20 points for writing. He has semantic paraphasias. |
| --- |
| **ChatGPT Answer:**  **Speech and Language Assessment Report**  **Client Information**  Name: Emre Kara  Age: 60 years  Language: Turkish  Evaluator: Fatma Uslu, Speech and Language Therapist  **Medical History**  Mr. Kara experienced a stroke affecting the left hemisphere middle cerebral artery due to hypertension approximately three months ago. This has resulted in right sided hemiparesis and aphasia. Post-stroke, he spent two days in an intensive care unit and was hospitalized for a total of 15 days. His vision and hearing capabilities are reported to be normal.  **Personal and Social History**  Mr. Kara is a retired teacher. He is married and has one child. His cooperation during the evaluation process was commendable.  **Cognitive Status**  Mr. Kara's Mini-Mental State Examination (MMSE) score is 26, indicating largely preserved cognitive functions.  **Motor Speech and Swallowing Assessment**  Evaluation for motor speech disorders was conducted and Mr. Kara showed no signs of dysarthria or apraxia of speech. His swallowing function is also reported to be normal.  **Aphasia Assessment**  The Turkish Aphasia Language Assessment Test (TALAT) was administered to evaluate Mr. Kara's language abilities. The following scores were obtained:  - Speaking fluency: 12  - Auditory comprehension: 50  - Repetition: 8  - Naming: 14  - Reading: 20  - Grammar: 8  - Speech actions: 8  - Writing: 20  These scores reflect notable deficits in speaking fluency, repetition, naming, grammar, speech actions, and to a lesser extent, in reading and writing.  **Observations**  Mr. Kara demonstrated semantic paraphasias during the evaluation, which is characterized by using words that are similar in meaning to the target words, but are not the correct words intended.  **Summary and Recommendations**  Mr. Kara is experiencing aphasia post-stroke, specifically in areas of speaking fluency, repetition, naming, grammar, speech actions, and to a lesser extent, reading and writing. His preserved cognitive functions and good cooperation present as positive factors for potential recovery. Considering the obtained findings, it is recommended that Mr. Kara engages in regular speech and language therapy. The therapy should particularly focus on areas where significant deficits have been identified, including speaking fluency, repetition, naming, and grammar. It is also suggested that family involvement could be beneficial in Mr. Kara's recovery process, providing emotional support and helping reinforce therapeutic strategies at home. Regular monitoring and re-evaluation of Mr. Kara's progress is advised to adjust the therapy plan as necessary. |

**3b) Aphasia -  Creating Assessment Material**

| **Prompt:**  Create auditory comprehension test for assessing aphasia. |
| --- |
| **ChatGPT Answer:**  Creating an auditory comprehension test for assessing aphasia can be a complex process that should ideally be developed and supervised by professionals in the field of speech-language pathology. However, I can give you a basic outline of what such a test might look like, incorporating elements of auditory comprehension that can often be challenging for individuals with aphasia.  **1. Single Word Comprehension:**     - Tester: "Point to the dog."     - Material: Pictures of a dog, a cat, and a car are laid out in front of the patient.  **2. Object Function Comprehension:**     - Tester: "Show me what you do with a pen."     - Material: A pen, a cup, and a pair of glasses are laid out in front of the patient.  **3. Simple Sentence Comprehension:**     - Tester: "Touch your nose after you clap your hands."     - Material: None, this is a test of action comprehension.  **4. Complex Sentence Comprehension:**     - Tester: "If it's sunny outside, point to the yellow square, otherwise point to the red circle."     - Material: A yellow square and a red circle are laid out in front of the patient.  **5. Auditory Discrimination:**     - Tester: "Is 'bat' the same as 'cat'?"     - Material: None, this is a test of phoneme discrimination.  **6. Contextual Understanding:**     - Tester: "What would you do if you saw a red light while driving?"     - Material: None, this is a test of comprehension of context and situation.  **7. Paragraph Comprehension:**     - Tester: Reads a short, simple story (e.g., "A girl named Jane has a big red ball. She likes to play with it at the park. One day, the ball rolls away and a dog brings it back to her.")     - Tester: "Who lost the ball? Where does Jane like to play? Who brought the ball back?"  Remember, this is just a very basic framework. Aphasia is a complex disorder with many variations and levels of severity, and a comprehensive assessment should cover a range of skills and be adjusted to the individual's abilities. Furthermore, understanding and answering these questions requires not only auditory comprehension but also other skills such as visual comprehension and motor response, which could be affected by the individual's condition.  This test should be administered by a trained professional who can correctly interpret the results and provide the appropriate feedback and treatment plan. Always consult with a healthcare provider for personalized medical advice. |

**3c) Aphasia - Clinical Decision Support**

| **Prompt:**  Support me in making clinical decision about the client whose detalis I will give you below.  The client's name is Emre Kara. He is a 60 years old Turkish patient who had a stroke in the left hemisphere middle cerebral artery of his brain due to hypertension three months ago. His vision and hearing is normal. There is a history of stay in intensive care unit for two days. He was hospitalized for 15 days. He was evaluated by Fatma Uslu, Speech and Language Therapist at the Speech and Language Therapy Unit of Türkiye University. He is married and he has one child. He was a teacher and he is retired. He has right sided hemiparesis and he has aphasia. His cooperation is good. His mini mental test score is 26. He was evaluated for motor speech disorder. He has not dysartria or apraxia of speech. His swallowing is normal. Turkish Aphasia Language Assessment Test (TALAT) was performed and according to test his scores was 12 points for speaking fluency, 50 points for auditory comprehension, 8 points for repetition, 14 points for naming, 20 points for reading, 8 points for grammar, 8 points for speech actions, and 20 points for writing. He has semantic paraphasias. |
| --- |
| **ChatGPT Answer:**  Based on the information provided, Emre Kara seems to have suffered from a stroke which has affected his language abilities, leading to a diagnosis of aphasia. His other functions such as vision, hearing, and swallowing are normal. He does not have dysarthria or apraxia of speech, motor speech disorders that cause issues with the physical production of speech.  His right sided hemiparesis (weakness on one side of the body) will likely require physical therapy, especially given his relatively high mini mental state examination score of 26, which suggests he has largely preserved cognitive function.  In terms of language therapy, the results from the Turkish Aphasia Language Assessment Test (TALAT) can help guide interventions. His scores indicate areas of strength and weakness:  - **Auditory comprehension (50 points)** and **writing (20 points)** appear to be his strongest language abilities. These areas can be capitalized on during therapy to help support his weaker areas.  - His **speaking fluency (12 points)**, **naming (14 points)**, and **reading (20 points)** indicate some level of difficulty, and may benefit from targeted therapeutic interventions.  - **Repetition (8 points)**, **grammar (8 points)**, and **speech actions (8 points)** are areas of considerable challenge. These will likely be primary targets in language therapy.  - The presence of **semantic paraphasias** (substituting one word for another related word, like saying "chicken" for "duck") suggests difficulties with word retrieval or language organization. This will also be an area to address in therapy.  Based on the above, an appropriate speech and language therapy intervention might include:  **1. Auditory Comprehension:** Since his comprehension skills are relatively strong, therapy sessions could utilize auditory instructions to help improve other language skills. For example, auditory cueing could be used to facilitate word retrieval for naming tasks.  **2. Naming and Reading:** Naming and reading tasks can be incorporated into therapy sessions to enhance his word-finding skills and improve reading comprehension. This can be particularly beneficial if Emre enjoys reading.  **3. Repetition, Grammar, and Speech Actions:** Since these are Emre's areas of greatest difficulty, targeted exercises to improve sentence structure and grammatical construction should be included. Given his profession as a teacher, these skills could be particularly meaningful to him.  **4. Semantic Paraphasias:** Working on word association games and other semantic exercises can help to target his word substitution issues.  **5. Use of Augmentative and Alternative Communication (AAC):** Given his relatively high scores in writing and auditory comprehension, Emre might benefit from AAC systems, which could involve writing or typing to communicate when speech is challenging.  **6. Family Involvement:** Engaging Emre's family in therapy, educating them about aphasia, and teaching them communication strategies will also be important for his recovery and will provide him with support outside of therapy.  As always, therapy should be tailored to Emre's individual interests and needs, and regular re-assessments should be carried out to ensure the therapy remains appropriate as his condition changes. Furthermore, close collaboration with other medical professionals involved in his care (like neurologists, physical therapists) will provide a more holistic care plan.  Remember to approach all decisions ethically, with consideration of Emre's personal preferences, cultural background, and right to autonomy. Emphasize a client-centered approach in which Emre actively participates in setting his therapeutic goals. |

**3d) Aphasia -  Creating Therapy Stimuli**

| **Prompt:**  Create a breakfast-themed Thematic Language Stimulation Therapy material to be used in aphasia therapy. |
| --- |
| **ChatGPT Answer:**  **Thematic Language Stimulation Therapy Material: Breakfast**  1. **Image Board:**     - Start by showing the patient a large image board that depicts a typical breakfast scene. It should include a variety of items such as a bowl of cereal, a cup of coffee, a glass of orange juice, slices of toast, eggs, bacon, pancakes, fruit, and a newspaper. Make sure the images are clear and identifiable.  2. **Vocabulary List:**     - Cereal     - Coffee     - Orange Juice     - Toast     - Egg (Scrambled, Boiled, Fried, Poached)     - Bacon     - Pancakes     - Fruit (Banana, Apple, Berries, Orange)     - Newspaper  3. **Description:**     - Begin by describing the image and the objects within it. For instance: "In the image, you see a table set for breakfast. There's a bowl of cereal, a steaming cup of coffee next to a glass of orange juice, and a plate of eggs and bacon. There are also pancakes on another plate, and some fruits are set to the side. A newspaper is spread open next to the meal."  4. **Discussion Questions:**     - What do you usually have for breakfast?     - Can you describe your favorite breakfast meal?     - What is the importance of breakfast?     - How do you like your eggs cooked?     - Do you drink coffee or tea in the morning?     - What fruits do you enjoy with your breakfast?  5. **Repetition Exercises:**     - Please repeat after me: "I am eating breakfast."     - Please repeat after me: "I enjoy coffee with my breakfast."     - Please repeat after me: "I like my eggs scrambled."  6. **Word Generation Exercises:**     - Name three types of cereal.     - Name three types of fruit you might eat at breakfast.     - Name three drinks you might have with breakfast.  7. **Sentence Generation Exercises:**     - "I like to have ____ for breakfast."     - "My favorite type of egg is ____."     - "I prefer to drink ____ in the morning."  8. **Role-play Scenarios:**     - Ordering breakfast at a restaurant.     - Making breakfast in the kitchen.     - Talking about breakfast preferences with a friend.  Remember, patience and encouragement are key. Allow the patient time to process and respond. Regular practice and repetition will help in language retrieval and usage. |

**3e) Aphasia - Therapy Planning**

| **Prompt:**  Create a speech and language therapy plan for the client with aphasia, whose information I will give you below:  The client's name is Emre Kara. He is a 60 years old Turkish patient who had a stroke in the left hemisphere middle cerebral artery of his brain due to hypertension three months ago. His vision and hearing is normal. There is a history of stay in intensive care unit for two days. He was hospitalized for 15 days. He was evaluated by Fatma Uslu, Speech and Language Therapist at the Speech and Language Therapy Unit of Türkiye University. He is married and he has one child. He was a teacher and he is retired. He has right sided hemiparesis and he has aphasia. His cooperation is good. His mini mental test score is 26. He was evaluated for motor speech disorder. He has not dysartria or apraxia of speech. His swallowing is normal. Turkish Aphasia Language Assessment Test (TALAT) was performed and according to test his scores was 12 points for speaking fluency, 50 points for auditory comprehension, 8 points for repetition, 14 points for naming, 20 points for reading, 8 points for grammar, 8 points for speech actions, and 20 points for writing. He has semantic paraphasias. |
| --- |
| **ChatGPT Answer:**  Speech and Language Therapy Plan for Emre Kara  **Client:** Emre Kara, 60-year-old retired teacher  **Presenting Issues:** Aphasia and right-sided hemiparesis following a left-hemisphere middle cerebral artery stroke  **Assessment:** Emre has been evaluated using the Turkish Aphasia Language Assessment Test (TALAT). He presents with semantic paraphasias, demonstrating good auditory comprehension, moderate speaking fluency, naming, reading, and writing skills, and more significant difficulties with repetition, grammar, and speech actions.  **Goals of Therapy:**  1. To improve overall communication ability and restore as much language function as possible  2. To enhance Emre's ability to participate in everyday conversations and social interactions  3. To boost his confidence and reduce anxiety related to speech issues  **Therapy Strategies and Techniques:**  **1. Errorless Learning Therapy:** This approach will be used to reinforce correct responses and reduce the instances of semantic paraphasias. We will use structured tasks to enhance Emre's vocabulary and ability to name objects and situations correctly.  **2. Semantic Feature Analysis Therapy:** This will help Emre increase his ability to name items by describing their features, group, function, action, and location. By categorizing words and using associative learning, we can improve his word-finding skills.  **3. Melodic Intonation Therapy:** This can be used to improve speech output and fluency by using melody and rhythm. Despite his difficulty with speech actions and grammar, Emre may find it easier to 'sing' phrases rather than speak them.  **4. Script Training Therapy:** We will work on common scripts (e.g., ordering food, greeting someone) to help Emre engage in daily conversations more fluently.  **5. Visual Communication Therapy:** This can support Emre's written and verbal communication by using visual aids like pictures and symbols.  **6. Cognitive-Communication Training:** To enhance cognitive aspects of communication, such as attention, memory, and problem-solving skills that will further benefit his language abilities.  **7. Reading and Writing Therapy:** To continue improving his skills in reading and writing, we will introduce exercises that align with his interests (like reading newspapers or writing letters) to maintain his motivation.  **Frequency of Sessions:** Therapy sessions will take place 2-3 times a week, initially, with the plan to adjust frequency based on progress and tolerance.  **Family/Caregiver Involvement:** Given Emre's good cooperation, we will involve his spouse and child in the therapy process. They can provide support during exercises, monitor Emre's progress, and foster a positive, encouraging environment.  **Monitoring Progress:** Emre's progress will be monitored throughout the course of therapy, and modifications will be made as necessary. Re-evaluation will occur every three months to update the treatment plan as required.  This speech and language therapy plan is tailored to Emre's needs and will be adapted over time to accommodate his progress and evolving goals. The aim is to help Emre regain his confidence and functionality in language to improve his quality of life post-stroke. |

**3f) Aphasia - Creating Education Material**

| **Prompt:**  Create educational materials for families to support individuals with Broca's aphasia. |
| --- |
| **ChatGPT Answer:**  **Broca's Aphasia: A Guide for Families**  Broca's aphasia, also known as expressive aphasia, is a condition characterized by difficulties with language production, though understanding of language is typically maintained. This educational guide provides some basic tips and strategies for family members to support their loved ones with Broca's aphasia.  **Understanding Broca's Aphasia**  Broca's aphasia is a type of non-fluent aphasia that results from damage to the frontal lobe of the brain, often due to stroke or brain injury. Individuals with this condition have difficulty with speech production, word-finding, and grammatical construction, though their comprehension of language is typically less affected.  **Communication Strategies for Families**  **1. Patience:** Understand that communication will take more time. Avoid rushing your loved one, and allow them time to express their thoughts.  **2. Simplify language:** Use simple sentences and words. This can lessen the cognitive load for your loved one, making communication more accessible.  **3. Validate effort, not just accuracy:** Acknowledge your loved one's efforts in communication, not just the correctness of the speech. This can help to encourage their participation in conversation.  **4. Non-verbal communication:** Encourage the use of non-verbal communication methods like gestures, facial expressions, or visual aids. A mix of communication modes can aid understanding and expression.  **5. Promote independence:** Encourage your loved one to communicate as independently as possible. Even if it takes longer, it’s important for their confidence and language recovery.  **Useful Tools and Resources**  **1. Aphasia apps:** There are various apps designed specifically for aphasia rehabilitation, such as Tactus Therapy, Constant Therapy, and Speech and Language Therapy for Aphasia (SALT). These can aid in speech therapy exercises.  **2. Visual aids:** Visual cues like picture boards or charts can be extremely useful in aiding communication.  **3. Support groups:** Local or online support groups for individuals with aphasia and their families can offer emotional support and practical advice from those who have had similar experiences.  **Professional Support**  Consider professional support options for your loved one, such as:  **1. Speech-Language Pathologist (SLP):** An SLP specializes in communication disorders and can provide tailored strategies and exercises to improve speech.  **2. Occupational Therapist (OT):** An OT can help your loved one learn new ways to approach daily tasks that have been affected by aphasia, such as using the phone or writing.  **3. Psychologist:** A psychologist can provide support in managing the emotional and psychological challenges associated with aphasia.  **Conclusion**  Supporting a loved one with Broca's aphasia can be challenging, but understanding the condition and implementing these strategies can greatly improve the quality of life for both the individual with aphasia and their families. Remember, patience, empathy, and a positive outlook can go a long way in supporting your loved one on their recovery journey. |

**4a) Stuttering -  Report Writing**

| **Prompt:**  Write a stuttering assessment report according to the information I will give you below.  The client's name is Selim Alkan. He is 9 years and 2 months old. He goes to 3rd grade. He applied to Türkiye University Speech and Language Therapy Unit for stuttering therapy. The assessment was made by speech and language therapist Fatma Uslu on March 7, 2023. Selim's general development is reported to be normal by his parents. His stuttering first appeared at the age of 4 with syllable repetitions. Selim's grandfather also stutters. According to Selim's parents, they believe that the onset of stuttering was possibly triggered by stress and sibling jealousy. Initially, Selim’s parents anticipated that the stuttering would resolve on its own with time. However, as the stuttering persisted for over a year, they sought therapy from a speech and language therapist, which lasted for three months. Selim's family noted an increase in his stuttering when he started first grade, correlating it with anxiety. Selim is described as a stubborn child who struggles with managing his emotions. He is an extroverted child who is well-liked by his peers. When he is stressed, nervous and excited, he stutters more and has difficulty expressing himself. His parents think that he is disturbed by his stuttering and are concerned about the process, but they have never talked to Selim about it. His parents do not react when he stutters. They want him to be able to manage his stuttering. Selim stutters in repetitions, prolongations, and blocks. During the assessment, Selim expressed his distress regarding his stuttering. Stuttering Severity Instrument-4 TR (SSI-4-TR) was administered to Selim. It was observed that his stuttering was at a severe level (SSI-4-TR total score 28). The frequency of stuttering observed during the assessment was 5% during the reading task and 8% during the speaking task. The average duration of stuttering moments was 2.5 seconds. Selim’s stuttering moments are accompanied by secondary behaviors such as pressing the lips, turning the head back, averting the eyes, and tapping the foot on the ground. |
| --- |
| **ChatGPT Answer:**  **STUTTERING ASSESSMENT REPORT**  **Client:** Selim Alkan  **Age:** 9 years 2 months  **Grade:** 3rd grade  **Assessment Date:** March 7, 2023  **Therapist:** Fatma Uslu  **Assessment Location:** Türkiye University Speech and Language Therapy Unit  **Background Information:**  Selim Alkan presented with a history of stuttering which commenced at the age of 4, reportedly with syllable repetitions. There is a familial history of stuttering (grandfather). His parents propose that his stuttering onset was possibly triggered by stress and sibling rivalry. The family initially anticipated spontaneous recovery, however, after observing the persistent nature of stuttering over a year, sought therapy. Therapy lasted for three months but Selim's stuttering increased when he started first grade, with a possible correlation to heightened anxiety.  Selim is generally described as a spirited and extroverted child, with some difficulty managing his emotions. His stuttering is observed to be more pronounced when he is stressed, nervous, or excited. His parents are aware of his distress about his stuttering but have not openly discussed it with him. They do not react overtly to his stuttering instances but wish for him to manage his stuttering better.  **Observations & Assessment:**  Selim stutters in repetitions, prolongations, and blocks. During the assessment, he expressed distress related to his stuttering. His stuttering was observed to be at a severe level on the Stuttering Severity Instrument-4 TR (SSI-4-TR), with a total score of 28. The frequency of stuttering was 5% during a reading task and 8% during a speaking task. The average duration of stuttering moments was 2.5 seconds. Accompanying secondary behaviors include pressing the lips, turning the head back, averting the eyes, and tapping the foot on the ground.  **Impressions & Recommendations:**  Selim demonstrates severe stuttering with both repetition and prolongation components, which appears to increase under stress and excitement. Secondary behaviors suggest an elevated level of discomfort and distress. The increased stuttering observed at the beginning of formal education also suggests that environmental factors may be exacerbating his stuttering.    Family education is recommended to provide the parents with strategies to support Selim in managing his stuttering. Further, an open discussion about stuttering may help Selim to better understand his speech patterns and reduce his distress. Continued speech and language therapy is recommended with a focus on reducing stuttering severity and improving fluency, as well as techniques for managing anxiety and emotion regulation.  Fatma Uslu  Speech and Language Therapist |

**4b) Stuttering -  Creating Assessment Material**

| **Prompt:**  Create clinical stuttering assessment instrument for adult people who stuttter. |
| --- |
| **ChatGPT Answer:**  **Adult Stuttering Assessment Instrument (ASAI)**  Section 1: Background and History  1.1 Age:  - Please state your current age:  1.2 Gender:  - Please state your gender:  1.3 Onset:  - At what age did you first notice the stuttering?  1.4 History:  - Has there been any improvement or worsening of the stuttering over time?  1.5 Family History:  - Is there a family history of stuttering?  Section 2: Current Stuttering Characteristics  2.1 Frequency:  - How often do you stutter? (Rarely, Sometimes, Frequently, Always)  2.2 Types of Disfluencies:  - Describe the types of disfluencies you experience. (Repetitions, Prolongations, Blocks)  2.3 Physical Accompaniments:  - Do you have any physical reactions when you stutter? (Eye blinking, Facial grimacing, Head nodding)  2.4 Secondary Behaviors:  - Are there any behaviors you engage in to avoid stuttering or to escape from stuttering once it has started? (Word substitution, Interjections, Circumlocutions)  Section 3: Communication Attitude  3.1 Feelings about Stuttering:  - How do you feel about your stuttering? (Neutral, Frustrated, Embarrassed, Anxious)  3.2 Impact on Communication:  - How does your stuttering affect your communication in social, academic, or occupational settings?  3.3 Avoidance Behaviors:  - Are there any specific situations or words that you tend to avoid due to your stuttering?  Section 4: Speech Sample Analysis  4.1 Sample Collection:  - Please provide a speech sample of at least 200 words. This could be in the form of spontaneous speech, reading, or narration.  4.2 Disfluency Count:  - The clinician will note the frequency of disfluencies, types of disfluencies, and the duration of each disfluency from the speech sample.  Section 5: Quality of Life  5.1 Impact on Daily Life:  - How does your stuttering affect your daily activities?  5.2 Emotional Impact:  - What emotional impact does your stuttering have on you? (E.g. stress, anxiety, depression)  Section 6: Goals for Therapy  6.1 Desired Outcomes:  - What are your desired outcomes from stuttering therapy?  6.2 Expectations:  - What are your expectations from the therapy process?  *Disclaimer: This assessment tool should be used by qualified speech and language therapists. It does not substitute a comprehensive evaluation and clinical judgement. If you are an individual who stutters, please consult with a licensed professional for assessment and treatment options.* |

**4c) Stuttering - Clinical Decision Support**

| **Prompt:**  Support me in making a clinical decision about the client whose details I have given below.  The client's name is Selim Alkan. He is 9 years and 2 months old. He goes to 3rd grade. He applied to Türkiye University Speech and Language Therapy Unit for stuttering therapy. The assessment was made by speech and language therapist Fatma Uslu on March 7, 2023. Selim's general development is reported to be normal by his parents. His stuttering first appeared at the age of 4 with syllable repetitions. Selim's grandfather also stutters. According to Selim's parents, they believe that the onset of stuttering was possibly triggered by stress and sibling jealousy. Initially, Selim’s parents anticipated that the stuttering would resolve on its own with time. However, as the stuttering persisted for over a year, they sought therapy from a speech and language therapist, which lasted for three months. Selim's family noted an increase in his stuttering when he started first grade, correlating it with anxiety. Selim is described as a stubborn child who struggles with managing his emotions. He is an extroverted child who is well-liked by his peers. When he is stressed, nervous and excited, he stutters more and has difficulty expressing himself. His parents think that he is disturbed by his stuttering and are concerned about the process, but they have never talked to Selim about it. His parents do not react when he stutters. They want him to be able to manage his stuttering. Selim stutters in repetitions, prolongations, and blocks. During the assessment, Selim expressed his distress regarding his stuttering. Stuttering Severity Instrument-4 TR (SSI-4-TR) was administered to Selim. It was observed that his stuttering was at a severe level (SSI-4-TR total score 28). The frequency of stuttering observed during the assessment was 5% during the reading task and 8% during the speaking task. The average duration of stuttering moments was 2.5 seconds. Selim’s stuttering moments are accompanied by secondary behaviors such as pressing the lips, turning the head back, averting the eyes, and tapping the foot on the ground. |
| --- |
| **ChatGPT Answer:**  Based on the information given, here are some recommendations to assist in the clinical decision-making process for Selim:  **1. Individual Speech Therapy:** Considering Selim's severe stuttering score on the SSI-4-TR and its impact on his day-to-day communication, individualized speech therapy should be initiated. The therapy could be based on techniques such as the Lidcombe Program, which is found effective in children under the age of 10. The program targets reducing the frequency of stuttering by means of positive reinforcement from parents when fluent speech is noted.  **2. Cognitive Behavioral Therapy (CBT):** In view of Selim's emotional regulation struggles, anxiety, and the increased stuttering under stress, Cognitive Behavioral Therapy (CBT) could be beneficial. CBT can provide him with coping mechanisms for anxiety and improve his emotional management, which could indirectly help control his stuttering.  **3. Parental Involvement:** It is essential to involve Selim's parents in the therapy process. They should be educated about stuttering and encouraged to discuss it with Selim in an appropriate and supportive manner. This can help to lessen any potential negative feelings or stigma associated with his stuttering.  **4. School Collaboration:** Collaboration with Selim's school may also be beneficial. Teachers can be made aware of Selim's condition, his treatment plan, and how they can support him in class. This can create a more understanding and accommodating environment for Selim, reducing possible stressors at school.  **5. Monitoring Progress:** Regular monitoring and follow-up should be conducted to assess progress and adjust therapy techniques as needed. Changes in the severity and characteristics of his stuttering, the impact of stuttering on his life, and his emotional well-being should be evaluated regularly.  **6. Stuttering Modification Therapy:** As Selim presents with secondary behaviors associated with his stuttering, he could benefit from stuttering modification therapy, designed to reduce these behaviors and the fear associated with stuttering.  **7. Group Therapy:** Considering that Selim is an extrovert and well-liked by his peers, involving him in group therapy can also be beneficial. It can provide a supportive environment where he can interact with other children facing similar challenges. This can potentially improve his self-esteem and decrease feelings of isolation or embarrassment associated with stuttering.  These are suggestions based on the provided case history. They need to be adapted based on Selim's response to therapy, the family's commitment and involvement, and Selim's changing needs as he grows older. Always remember, the goal should not only be to reduce stuttering but also to improve Selim's overall communication confidence and emotional well-being. |

**4d) Stuttering - Creating Therapy Stimuli**

| **Prompt:**  Create 100 Turkish one, two and three syllable words for using in the fluency shaping therapy with an adult client. |
| --- |
| **ChatGPT Answer:**  Sure, here are 100 Turkish words varying from one to three syllables.  One syllable words:  1. Ben  2. Sen  3. Göz  4. Kul  5. El  6. Ay  7. Dal  8. Öz  9. Yüz  10. Taş  11. Dök  12. Yüz  13. Yaz  14. Top  15. Saç  16. Can  17. Kırl  18. Yağ  19. Koş  20. Renk  21. Kök  22. Denk  23. Alt  24. Yük  25. Kırmızı  26. Gök  27. Yan  28. Bahçe  29. İş  30. Kal  31. Bir  32. İki  33. Kaş  34. Kav  35. Kez  Two syllable words:  36. Bahar  37. Umut  38. Fikir  39. Sanat  40. Anlam  41. Tatil  42. İlkbahar  43. Merhaba  44. Bilgi  45. Tabak  46. Nefret  47. Yıldız  48. Kahve  49. Tebrik  50. Çalış  51. Yeşil  52. Tarla  53. İstek  54. Belge  55. Üzüm  56. Masal  57. Yazılı  58. Yıldız  59. Dans  60. Kesim  61. Yarat  62. Muhalefet  63. Kırlent  64. Bilgisayar  65. Öğrenci  66. Kalabalık  67. Gazete  68. Hayvan  Three syllable words:  69. Çikolata  70. Yayınevi  71. Arabistan  72. Cumartesi  73. Bilinçaltı  74. Ayakkabı  75. Türkiye  76. Çocukluk  77. İstanbul  78. Kırmızı  79. Tebrikler  80. Sınırsız  81. Üzgünüm  82. Doğumgünü  83. İskeleye  84. Felsefe  85. Şemsiye  86. İhtiyacımız  87. Genişlik  88. Ayçiçeği  89. Görüşmek  90. Çeşitli  91. Hayalperest  92. Kitaplık  93. Cumhuriyet  94. Bilgisayarım  95. Eğlence  96. Papatya  97. Müzikal  98. Elektronik  99. Organik  100. Çalışkan  These words range in difficulty and can be used in therapy to help build fluency and confidence in the client's ability to articulate different sounds and syllables. |

**4e) Stuttering - Therapy Planning**

| **Prompt:**  Create a stuttering therapy plan for the person who stutter, with given information below.  The client's name is Selim Alkan. He is 9 years and 2 months old. He goes to 3rd grade. He applied to Türkiye University Speech and Language Therapy Unit for stuttering therapy. The assessment was made by speech and language therapist Fatma Uslu on March 7, 2023. Selim's general development is reported to be normal by his parents. His stuttering first appeared at the age of 4 with syllable repetitions. Selim's grandfather also stutters. According to Selim's parents, they believe that the onset of stuttering was possibly triggered by stress and sibling jealousy. Initially, Selim’s parents anticipated that the stuttering would resolve on its own with time. However, as the stuttering persisted for over a year, they sought therapy from a speech and language therapist, which lasted for three months. Selim's family noted an increase in his stuttering when he started first grade, correlating it with anxiety. Selim is described as a stubborn child who struggles with managing his emotions. He is an extroverted child who is well-liked by his peers. When he is stressed, nervous and excited, he stutters more and has difficulty expressing himself. His parents think that he is disturbed by his stuttering and are concerned about the process, but they have never talked to Selim about it. His parents do not react when he stutters. They want him to be able to manage his stuttering. Selim stutters in repetitions, prolongations, and blocks. During the assessment, Selim expressed his distress regarding his stuttering. Stuttering Severity Instrument-4 TR (SSI-4-TR) was administered to Selim. It was observed that his stuttering was at a severe level (SSI-4-TR total score 28). The frequency of stuttering observed during the assessment was 5% during the reading task and 8% during the speaking task. The average duration of stuttering moments was 2.5 seconds. Selim’s stuttering moments are accompanied by secondary behaviors such as pressing the lips, turning the head back, averting the eyes, and tapping the foot on the ground. |
| --- |
| **ChatGPT Answer:**  Stuttering Therapy Plan for Selim Alkan  Based on the evaluation of Fatma Uslu, Selim Alkan's stuttering presents at a severe level. Selim demonstrates behaviors such as repetitions, prolongations, blocks, and secondary behaviors. The therapy plan will address these symptoms and help Selim manage his stuttering effectively.  **1. Stuttering Modification Therapy:**  Stuttering modification therapy aims to modify the stuttering to make it easier and less tense. Selim will learn to stutter in a more relaxed way through the four stages:     a. Identification: Selim will be taught to identify the physical sensations and situations that exacerbate his stuttering.     b. Desensitization: Selim will be exposed gradually to stuttering situations to help him become less sensitive to stuttering.     c. Modification: Techniques such as pull-outs and cancellations will be used to modify Selim's stuttering moments.     d. Stabilization: Selim will learn to generalize the techniques learned in therapy across all speaking situations.  **2. Cognitive Behavioral Therapy (CBT):**  Given that Selim's stuttering is affected by his emotions, CBT can be used to manage his anxiety and stress levels. CBT will focus on:     a. Understanding and managing anxiety: Selim will be guided to understand the feelings and thoughts that increase his anxiety and contribute to his stuttering.     b. Developing coping strategies: Techniques such as deep breathing, mindfulness, and relaxation exercises will be introduced to help Selim manage his emotions better.  **3. Fluency Shaping Therapy:**  Fluency shaping therapy will help Selim develop new speaking patterns that produce less stuttering. Techniques that will be used include:     a. Slow and controlled speech: Selim will learn to slow his speech down and control his breathing while speaking.     b. Gradual increase in speech rate: As Selim becomes comfortable with slow speech, the speed will be gradually increased while maintaining fluency.  **4. Family Training:**  Training Selim's family to support him is crucial. The family will be guided on:     a. Understanding stuttering: Selim's parents will be educated about stuttering, its nature, and how to respond to it.     b. Developing a supportive environment: Selim's family will be guided on how to provide a supportive and low-stress environment, which includes reducing demands on speech and minimizing criticism.     c. Communication strategies: The family will be guided on effective communication strategies such as giving Selim enough time to speak and not finishing his sentences for him.  **5. School Support:**  As Selim's stuttering increased when he started first grade, collaboration with his school is essential. The speech therapist will:     a. Educate his teachers about stuttering and how to support Selim in the classroom.     b. Collaborate with the school's special education team to ensure that Selim's needs are met.  **6. Self-Awareness and Acceptance:**  As Selim is affected by his stuttering, helping him understand and accept it is crucial. He will be guided to:     a. Express his feelings and thoughts about stuttering.     b. Develop a positive attitude towards his speech and himself.     c. Understand that it is okay to stutter and it does not define his abilities or worth.  Therapy will be evaluated every 3 months to assess the progress and make necessary changes. This therapy plan aims to not only improve Selim's fluency but also help him gain confidence and resilience. |

**4f) Stuttering - Creating Education Material**

| **Prompt:**  Create client education material for school-age stuttering. |
| --- |
| **ChatGPT Answer:**  **Understanding and Addressing School-Age Stuttering: A Guide for Parents and Caregivers**  **What is Stuttering?**  Stuttering, also known as stammering, is a communication disorder characterized by disruptions or disfluencies in a person's speech. It typically begins in childhood, often between ages two and six. It is characterized by repetitions (like "I-I-I want"), prolongations (like "ssssoup"), or blocks (complete stoppage of sound) that last more than usual. Additionally, physical tension or struggle behaviors may be associated with stuttering.  **How is Stuttering Different from Normal Disfluencies?**  Every child may have some speech disfluencies, especially when they are learning to speak. However, stuttering differs from these normal speech interruptions because of its frequency, duration, and the presence of secondary behaviors (like eye blinking or looking to the side). If a child stutters, these disfluencies are often more frequent and longer-lasting.  **Common Signs of Stuttering**  These signs can help you identify stuttering in school-age children:  1. Frequent repetition of sounds, syllables, or words, e.g., "c-c-can I have a c-c-c-cookie?"  2. Prolonged sounds, e.g., "ssssoup for dinner?"  3. Speech blocks or pause within a word.  4. Tension, struggling behaviors, or "speech motor 'freeze ups'"  5. Avoidance behaviors such as substituting words or not speaking.  6. Negative reactions to stuttering, such as frustration or embarrassment.  **How Can We Help Children Who Stutter?**  Stuttering can impact a child's academic performance, social interactions, and self-esteem. Therefore, it's essential to address stuttering in a supportive and understanding manner.  **1. Speech-Language Therapy:** A speech-language pathologist (SLP) can provide therapy to improve speech fluency and communication skills. SLPs can also teach strategies to navigate through stuttering moments more easily.  **2. Supportive Environment:** Encourage an open, positive, and patient communication environment at home and school. Try not to interrupt or complete sentences for the child, allowing them to finish their thoughts at their own pace.  **3. Education:** Teach the child, and those around them, about stuttering. This can reduce negative reactions, misunderstandings, and bullying.  **4. Emotional Support:** Encourage self-esteem and confidence in the child. Celebrate their strengths and achievements to minimize the focus on their speech.  **Useful Strategies for Parents and Teachers**  Here are some strategies you can apply to help your child:  **1. Listen patiently:** Be attentive to what your child is saying rather than how they are saying it.  **2. Speak Slowly:** Model slow, relaxed speech when talking with your child. This can help to reduce their speech pressure.  **3. Avoid Hurrying the Child:** Never rush a child who stutters to finish their thoughts or sentences. Provide them ample time to express themselves.  **4. Reduce Questions:** Sometimes, too many questions can pressure the child to speak. Engage them in more casual, comment-based conversations.  **5. Talk About Stuttering:** It's okay to talk about stuttering with your child. Discussing it can decrease its mystery and associated fear.  **6. Team Up With School:** Make sure the school is aware of your child's situation and is providing necessary support. Collaborate with teachers to ensure a positive and supportive environment.  Stuttering is not a choice or a result of nervousness. With proper guidance, understanding, and professional help, school-age children who stutter can express themselves confidently and navigate their daily lives more easily. |

**5a) Childhood Language Disorders - Report Writing**

| **Prompt:**  Write a speech and language assessment report according to the information I will give you below.  The client's name is Eren Duru. Eren is currently 4 years and 2 months old. A Speech and Language assessment was conducted at the Speech and Language Therapy Unit of Türkiye University on April 19, 2023. The assessment was carried out by Fatma Uslu, a speech and language therapist. Eren's early language development included the onset of first words at 24 months, and his motor skills are within the normal range. However, Eren's mother reported difficulty communicating with him and noticed similar challenges with his interaction among peers. Eren exhibits difficulties in both understanding and expressing language. He currently has a vocabulary of approximately 200 words and he often uses 2-3 word sentences and telegraphic speech. Furthermore, Eren struggles with following instructions and responding to questions appropriately. Hearing screening results indicate that Eren does not have any hearing-related issues. Additionally, the Leiter Non-verbal IQ test results suggest that Eren's non-verbal intelligence falls within the normal range compared to his peers. Eren's language skills were further evaluated using the Test of Early Language Development Turkish (TELD 3:T). According to the TELD 3:T results, Eren's spoken language index score was 69 (Degree of disorder: Very Weak); receptive language standard score of 87 (Degree of disorder: Below Average); expressive language standard score was found to be 61 (Degree of disorder: Very Weak). Eren is in the 2% segment. Regarding other potential risk factors such as neurological, genetic, and monolingual aspects, Eren's assessment did not reveal any abnormalities or concerns. |
| --- |
| **ChatGPT Answer:**  **Speech and Language Assessment Report**  **Client Information:**  Client Name: Eren Duru  Age: 4 years, 2 months  Date of Assessment: April 19, 2023  Location: Speech and Language Therapy Unit, Türkiye University  Assessor: Fatma Uslu, Speech and Language Therapist  **Background Information:**  Eren's language development onset of first words was at 24 months, which is at the later end of the typical range. His motor skills development appears to be within the normal range. Concerns were raised by his mother regarding difficulties in communicating with him and observed challenges with his interaction with peers.  **Presenting Difficulties:**  Eren exhibits difficulties in both receptive and expressive language, which includes understanding and expressing language. He currently has a vocabulary of approximately 200 words and often uses 2-3 word sentences and telegraphic speech. Eren struggles with following instructions and responding to questions appropriately.  **Assessment Tools and Results:**  **1. Hearing Screening:** Eren's hearing is within the normal range.  **2. Leiter Non-verbal IQ Test:** Eren's non-verbal intelligence falls within the normal range for his age.  **3. Test of Early Language Development Turkish (TELD 3:T):** Eren's performance is as follows:     - Spoken language index score: 69 (Degree of disorder: Very Weak)     - Receptive language standard score: 87 (Degree of disorder: Below Average)     - Expressive language standard score: 61 (Degree of disorder: Very Weak)  Eren is in the 2% segment, indicating significant challenges in his language skills compared to his peers.  **Additional Factors:**  Assessment did not reveal any other potential risk factors such as neurological, genetic, or monolingual factors contributing to Eren's speech and language difficulties.  **Summary:**  Eren exhibits significant difficulties in both receptive and expressive language abilities. This is consistent with his mother's reported observations of Eren's difficulty in communicating and interacting with peers. Eren's hearing and non-verbal cognitive abilities were found to be within the normal range, indicating that his language challenges are not due to hearing issues or overall cognitive ability. Further support and interventions are necessary to assist Eren in developing his language skills.  **Recommendations:**  Given the assessment findings, it is recommended that Eren receive speech and language therapy services to address his identified areas of need. Collaborative planning with his family and any involved professionals will be beneficial in creating an individualized intervention plan for Eren. Continual monitoring of his progress will be essential to assess the effectiveness of the intervention and make necessary modifications.  **Follow-Up:**  A review assessment will be scheduled in six months to monitor Eren's progress and adjust the treatment plan as necessary.  Fatma Uslu  Speech and Language Therapist  Türkiye University  Date: April 19, 2023 |

**5b) Childhood Language Disorders - Creating Assessment Material**

| **Prompt:**  Create assessment tasks for a 5 year and 3 months old child’s receptive language skills. |
| --- |
| **ChatGPT Answer:**  Assessment Task 1: Understanding Basic Instructions  - The child will be given simple one-step instructions such as "Touch your nose" or "Point to the window". Observe whether the child understands and follows these instructions.  Assessment Task 2: Comprehension of Prepositions  - Prepare an assortment of small toys and a toy box. Instruct the child to "Put the bear in the box" or "Place the car next to the box". This evaluates the child's understanding of prepositions such as 'in', 'on', 'under', 'next to'.  Assessment Task 3: Recognition of Colors and Shapes  - Show the child flashcards with different colors and shapes. Ask questions like "Which one is the red square?" or "Can you point to the blue circle?"  Assessment Task 4: Following a Two-Step Instruction  - Give the child two-step instructions to see if they can remember and execute them. For example, "Pick up the doll and put it on the chair".  Assessment Task 5: Comprehension of Basic Stories  - Read a short, age-appropriate story to the child. Afterward, ask simple questions about the story such as "Who was the main character?" or "Where did the story take place?" to assess the child's understanding.  Assessment Task 6: Understanding Opposites  - Provide the child with pairs of opposite words (e.g., hot/cold, big/small, happy/sad). Show them objects or images representing these concepts, and ask them to match the word to the correct object or image.  Assessment Task 7: Identifying Emotions  - Show the child pictures of people exhibiting different emotions (happy, sad, scared, surprised, etc.). Ask the child to identify the emotion in each picture.  Remember, the aim is to create a supportive environment where the child feels comfortable. Encourage the child and offer positive feedback, but do not correct or instruct during the assessment as this might interfere with the results. |

**5c) Childhood Language Disorders - Clinical Decision Support**

| **Prompt:**  Support me in making clinical decision about the client whose detalis I have given below.  The client's name is Eren Duru. Eren is currently 4 years and 2 months old. A Speech and Language assessment was conducted at the Speech and Language Therapy Unit of Türkiye University on April 19, 2023. The assessment was carried out by Fatma Uslu, a speech and language therapist. Eren's early language development included the onset of first words at 24 months, and his motor skills are within the normal range. However, Eren's mother reported difficulty communicating with him and noticed similar challenges with his interaction among peers. Eren exhibits difficulties in both understanding and expressing language. He currently has a vocabulary of approximately 200 words and he often uses 2-3 word sentences and telegraphic speech. Furthermore, Eren struggles with following instructions and responding to questions appropriately. Hearing screening results indicate that Eren does not have any hearing-related issues. Additionally, the Leiter Non-verbal IQ test results suggest that Eren's non-verbal intelligence falls within the normal range compared to his peers. Eren's language skills were further evaluated using the Test of Early Language Development Turkish (TELD 3:T). According to the TELD 3:T results, Eren's spoken language index score was 69 (Degree of disorder: Very Weak); receptive language standard score of 87 (Degree of disorder: Below Average); expressive language standard score was found to be 61 (Degree of disorder: Very Weak). Eren is in the 2% segment. Regarding other potential risk factors such as neurological, genetic, and monolingual aspects, Eren's assessment did not reveal any abnormalities or concerns. |
| --- |
| **ChatGPT Answer:**  Based on the data provided about Eren, he appears to be exhibiting symptoms of a Speech and Language Disorder, specifically Expressive and Receptive Language Disorder. The low scores on the TELD 3:T, especially the expressive language score, combined with difficulties following instructions, responding to questions, and general struggles in understanding and expressing language support this diagnosis.  The primary recommendations for Eren are as follows:  **1. Speech and Language Therapy:** Eren would benefit from direct intervention from a speech and language therapist to address his difficulties in both understanding and expressing language. The therapy should focus on enhancing Eren's receptive language abilities (understanding spoken language and following instructions) and his expressive language skills (formulating sentences, expanding vocabulary, and improving the complexity of his sentences). The therapy could also include working on social communication skills, given the reported difficulties interacting with peers.  **2. Parental Training:** It is also crucial to involve the family in Eren's treatment process. Parental training sessions, where Eren's parents are taught strategies to facilitate their child's language development at home, can be very beneficial. Strategies might include providing a language-rich environment, encouraging communication throughout daily activities, and modeling appropriate language use.  **3. Peer Interaction:** Encouraging interactions with peers can enhance Eren's social skills and language development. Enrolling Eren in a playgroup or a preschool setting (if not already done) may be helpful.  **4. Regular Review and Assessment:** Given the age and developmental stage of Eren, it's crucial to reassess his language skills at regular intervals (every 6 months, for example) to monitor his progress and adjust treatment strategies as necessary.  **5. Multidisciplinary Collaboration:** While Eren's initial assessments did not reveal concerns in other areas, maintaining collaboration with other professionals like pediatricians, psychologists, and educators can ensure a holistic approach to Eren's care. They can help monitor his progress and identify any other areas of need that may arise as he grows.  Remember, early intervention is key in managing speech and language disorders, so the earlier these strategies are implemented, the better for Eren's development. |

**5d) Childhood Language Disorders - Creating Therapy Stimuli**

| **Prompt:**  Create Focused Stimulation language therapy stimuli to target plural suffix. |
| --- |
| **ChatGPT Answer:**  Sure, here are several stimuli that could be used in a language therapy session to target the plural suffix.  **1. Picture Identification:**      - Present a series of pictures to the client. The first picture could be of one dog, the second picture could be of two dogs. Ask the client to identify each picture. If they identify the second picture as "two dog," gently correct them by saying, "two dogs." Repeat with different images.  **2. Sentence Completion:**      - Read out sentences to the client that require them to fill in the blanks with the correct form of the word. For example, "I have one cat. You have two __?"  **3. Story Reading:**      - Read a short story to the client that includes several instances of plural words. After each sentence, ask the client to repeat it. Correct any misuses of the plural form. For example, "The three pigs built their houses."  **4. Word Pairings:**      - Say a word in its singular form and ask the client to provide the plural form. For instance, "If one is a car, what are two?"  **5. Interactive Games:**      - Play a game like "I Spy" but only look for groups of things, asking the client to identify them using the plural form. For instance, "I spy with my little eye... trees."  **6. Memory Recall:**      - Ask the client to list from memory all the plural words they can think of in one minute. This will help not only stimulate their recall abilities, but also practice using the correct plural form.  **7. Visual Presentation:**      - Show the client an array of objects and ask them to count and identify what they see. This gives them a chance to practice using plural suffixes in a natural context.  Remember, therapy should always be tailored to the individual's needs and abilities, so these activities may need to be modified based on the client's current language abilities and goals. Always provide positive reinforcement and gentle correction to support the learning process. |

**5e) Childhood Language Disorders - Therapy Planning**

| **Prompt:**  Create a speech and language therapy plan for the client with language and communication needs, whose information I have given below:  The client's name is Eren Duru. Eren is currently 4 years and 2 months old. A Speech and Language assessment was conducted at the Speech and Language Therapy Unit of Türkiye University on April 19, 2023. The assessment was carried out by Fatma Uslu, a speech and language therapist. Eren's early language development included the onset of first words at 24 months, and his motor skills are within the normal range. However, Eren's mother reported difficulty communicating with him and noticed similar challenges with his interaction among peers. Eren exhibits difficulties in both understanding and expressing language. He currently has a vocabulary of approximately 200 words and he often uses 2-3 word sentences and telegraphic speech. Furthermore, Eren struggles with following instructions and responding to questions appropriately. Hearing screening results indicate that Eren does not have any hearing-related issues. Additionally, the Leiter Non-verbal IQ test results suggest that Eren's non-verbal intelligence falls within the normal range compared to his peers. Eren's language skills were further evaluated using the Test of Early Language Development Turkish (TELD 3:T). According to the TELD 3:T results, Eren's spoken language index score was 69 (Degree of disorder: Very Weak); receptive language standard score of 87 (Degree of disorder: Below Average); expressive language standard score was found to be 61 (Degree of disorder: Very Weak). Eren is in the 2% segment. Regarding other potential risk factors such as neurological, genetic, and monolingual aspects, Eren's assessment did not reveal any abnormalities or concerns. |
| --- |
| **ChatGPT Answer:**  **Speech and Language Therapy Plan for Eren Duru**  **I. Goal of Therapy:**  To improve Eren's language and communication skills with a focus on enhancing his receptive and expressive language abilities.  **II. Objectives:**  1. Enhance Eren's understanding and use of vocabulary.  2. Improve Eren's ability to follow instructions and respond to questions appropriately.  3. Foster development of sentence structure and grammar.  4. Improve Eren's ability to engage in social communication with peers and family.  **III. Intervention Plan:**  **A. Vocabulary Development:**  **1. Frequency:** 3 sessions per week  **2. Methods:** Utilize picture books, visual aids, real-life objects, and repetitive play to help Eren learn and remember words. Model correct language use during play and daily activities.  **B. Following Instructions and Response to Questions:**  **1. Frequency:** 2 sessions per week  **2. Methods:** Incorporate fun activities where Eren is given simple, then gradually more complex, instructions to follow. Use a variety of question types, starting with yes/no and moving to open-ended questions.  **C. Sentence Structure and Grammar:**  **1. Frequency:** 3 sessions per week  **2. Methods:** Work on extending Eren's sentences from 2-3 words to full sentences. Play-based activities, storytelling, and picture descriptions can be used. Emphasize correct grammar during daily conversations and activities.  **D. Social Communication Skills:**  **1. Frequency:** 1 session per week  **2. Methods:** Encourage interaction with peers in structured playgroups. Model appropriate communication behaviors, such as turn-taking, making eye contact, and appropriate use of gestures.  **IV. Evaluation:**  Re-evaluate Eren's language skills using the TELD 3:T after six months of therapy to measure improvement and adjust the therapy plan as necessary. Regular informal assessments should also be conducted to monitor progress.  **V. Parental Involvement:**  Parents are encouraged to reinforce strategies used in therapy during daily routines at home. Consistent use of these strategies is likely to expedite Eren's progress.  Please note that this plan is subject to modification based on Eren's unique needs and responses to intervention. Therapy should always be a collaborative process between the speech-language pathologist, the child, and the family. Regular communication about Eren's progress and any changes in his communication skills or behavior will be crucial for the success of this plan. |

**5f) Childhood Language Disorders - Creating Education Material**

| **Prompt:**  Create parent education materials for families of children with developmental language disorders. |
| --- |
| **ChatGPT Answer:**  **Navigating Developmental Language Disorders: A Guide for Parents**  **Introduction**  Raising a child with a Developmental Language Disorder (DLD) presents unique challenges. However, it's important to remember that your child can still lead a full, successful life. This guide aims to help parents understand DLD, its implications, and the role they can play in their child's development.  **Understanding Developmental Language Disorders**  DLD is a common childhood disorder affecting approximately 7% of individuals. It's characterized by difficulties with learning and using language, unrelated to hearing loss, general developmental delays, or other cognitive impairments.  **Early Signs and Symptoms**  Understanding the signs and symptoms of DLD can help with early intervention. Symptoms might include:  - Delay in reaching language milestones  - Difficulty following directions  - Struggling to find the right words  - Difficulty with sentence structure  - Issues with understanding questions or complex sentences  - Problems telling stories or relaying information  **Evaluation and Diagnosis**  If you suspect your child has DLD, the first step is a comprehensive evaluation. A certified Speech-Language Pathologist (SLP) will assess your child's receptive and expressive language skills, along with other aspects of communication.  **Treatment and Intervention**  DLD is a lifelong condition, but with early intervention and consistent therapy, your child can improve their language skills and quality of life. Therapies might include:  **1. Speech and Language Therapy:** This therapy focuses on improving your child's language skills and may involve various techniques and exercises.  **2. Social Skills Training:** As language is crucial for social interaction, this training assists children with DLD to navigate social scenarios.  **3. Educational Support:** This involves tailored learning strategies to help your child succeed academically.  **The Role of Parents and Family**  As a parent, you play a crucial role in your child's language development.  **1. Consistent Communication:** Engage your child in conversation regularly. Ask open-ended questions that encourage more than a one-word response.  **2. Language-rich Environment:** Read together, play word games, and narrate everyday activities.  **3. Patience and Understanding:** Give your child time to express themselves. Show understanding and offer gentle corrections when needed.  **4. Consistency with Therapy:** Consistently follow through with any home activities recommended by the SLP.  **Supporting Your Child in School**  Advocate for your child's needs at school. Collaborate with teachers to ensure your child is receiving the necessary accommodations.  **Joining a Support Network**  Connecting with other parents who have children with DLD can provide emotional support and practical advice.  **Conclusion**  Remember, every child with DLD is unique. Your love, patience, and commitment, combined with professional help, will ensure your child thrives.  For more resources on DLD:  - The American Speech-Language-Hearing Association (ASHA)  - The Communication Trust  - The DLD Project  Please consult with your healthcare professional or a certified Speech-Language Pathologist for personal advice and treatments. |

**6a) Voice Disorders - Report Writing**

| **Prompt:**  Write a voice assessment report according to the information I will give you below.  The client’s name is Sevim Uzun. Sevim is 35 years old. Her voice assessment was carried out at Türkiye University Speech and Language Therapy Unit on 29 April 2023. Sevim was assessed by the speech and language therapist Fatma Uslu. She smokes half a pack of cigarettes a day and she does not using alcohol. Sevim has never undergone surgery before and she has no history of allergies. Sevim, who does not have reflux, does not have a chronic disease that requiring regular medication. Sevim uses her voice intensively for about 6 hours a day and she is a primary school teacher. She has complaint of hoarseness, which started 1.5 months ago and progressed. Sevim drinks an average of 5 cups of tea and 2 cups of coffee a day. Sevim's score in the Voice Handicap Index-10 is 26. Her score on the Voice-Related Quality of Life scale is 27.  Sevim has a perceptually hoarse, breathy and rough voice, with a maximum phonation duration of 9 seconds and a s/z ratio of 1.6. Her GRBAS score is 7. Videolaryngostroboscopic examination revealed bilateral nodules in the anterior 1/3 of her vocal folds. |
| --- |
| **ChatGPT Answer:**  **VOICE ASSESSMENT REPORT**  **Patient Details:**  Name: Sevim Uzun  Age: 35 years old  Occupation: Primary school teacher  **Assessment Details:**  Date: 29 April 2023  Location: Türkiye University Speech and Language Therapy Unit  Assessed by: Fatma Uslu, Speech and Language Therapist  **Medical History:**  Tobacco Use: Half a pack of cigarettes a day  Alcohol Use: None  Previous Surgical Interventions: None  Allergies: None  Chronic Diseases: None  Gastroesophageal Reflux Disease (GERD): None  Medication Use: None  **Lifestyle Factors:**  Daily Voice Use: Approximately 6 hours  Beverage Consumption: 5 cups of tea and 2 cups of coffee per day  **Voice Complaint:**  The client has been experiencing hoarseness for the past 1.5 months, with a progressive severity.  **Assessment Scores:**  Voice Handicap Index-10: 26  Voice-Related Quality of Life: 27  **Voice Characteristics:**  Perceptually, Sevim's voice is hoarse, breathy, and rough. Her maximum phonation duration is 9 seconds and her s/z ratio is 1.6. This suggests an abnormal breath support and voice control, commonly associated with voice disorders.  **Objective Measures:**  GRBAS score: 7, indicating noticeable abnormalities in Grade of hoarseness, Roughness, Breathiness, Asthenia (weakness), and Strain.  **Physical Examination:**  Videolaryngostroboscopic examination revealed bilateral nodules in the anterior 1/3 of Sevim's vocal folds. These nodules are typically caused by overuse or misuse of the voice and can contribute to the observed voice characteristics and symptoms.  **Recommendations:**  Sevim is recommended to attend voice therapy sessions aimed at reducing vocal strain and improving vocal habits, potentially including less use of the voice, voice relaxation exercises, and hydration strategies. Her tobacco use may also be a contributing factor to her voice condition, and cessation could improve her vocal health. Further evaluation and follow-ups should be scheduled to monitor the progression of her condition and the effectiveness of the therapy.  **Signatory**  Fatma Uslu, Speech and Language Therapist  Türkiye University Speech and Language Therapy Unit  Date: 29 April 2023 |

**6b) Voice Disorders - Creating Assessment Material**

| **Prompt:**  Create a Turkish reading passage to evaluate hard glottal attacks of a client with voice disorder. |
| --- |
| **ChatGPT Answer:**  Sure, here's a reading passage designed to include a high frequency of words that start with vowels, causing potential for hard glottal attacks. These words are often preceded by pauses, which increases the likelihood of this type of voice production.    "Ahmet, işten eve varır varmaz, en sevdiği armut ağacının altına oturdu. Her gün aynı adımı atıyordu. İlk olarak o güzel ağacın altında, akşamın ilk anılarıyla baş başa kalıyordu. Armutlarını, ancak tatil günlerinde yiyordu. Oturduğu yerden aya bakıp, aşkın ve hayallerin dünyasına daldı.  Önce annesinin ev yapımı aşureyi düşündü. Annesi o kadar iyi aşure yapardı ki, Ahmet'in her an canı çekerdi. Arada bir annesi, en özel kıyafetlerini giyerek aşure pişirirdi.  Aşağı yukarı yarım saat oturduktan sonra, Ahmet içeriye girdi ve avluya baktı. Evin ağır kapısını açtı ve ayakkabılarını çıkararak evin içine girdi. Anahtarını asıp, annesiyle akşam yemeği için hazırlıklara başladı."  Remember, glottal attacks can cause strain and fatigue on the vocal cords. If your client is showing signs of discomfort or fatigue, it's important to take breaks and reassess. |

**6c) Voice Disorders - Clinical Decision Support**

| **Prompt:**  Support me in making a clinical decision about the client whose details I have given below.  The client’s name is Sevim Uzun. Sevim is 35 years old. Her voice assessment was carried out at Türkiye University Speech and Language Therapy Unit on 29 April 2023. Sevim was assessed by the speech and language therapist Fatma Uslu. She smokes half a pack of cigarettes a day and she does not using alcohol. Sevim has never undergone surgery before and she has no history of allergies. Sevim, who does not have reflux, does not have a chronic disease that requiring regular medication. Sevim uses her voice intensively for about 6 hours a day and she is a primary school teacher. She has complaint of hoarseness, which started 1.5 months ago and progressed. Sevim drinks an average of 5 cups of tea and 2 cups of coffee a day. Sevim's score in the Voice Handicap Index-10 is 26. Her score on the Voice-Related Quality of Life scale is 27.  Sevim has a perceptually hoarse, breathy and rough voice, with a maximum phonation duration of 9 seconds and a s/z ratio of 1.6. Her GRBAS score is 7. Videolaryngostroboscopic examination revealed bilateral nodules in the anterior 1/3 of her vocal folds. |
| --- |
| **ChatGPT Answer:**  Based on the information provided, Sevim Uzun has a vocal pathology which might be causing her hoarseness and voice change. Bilateral vocal fold nodules, as evidenced by videolaryngostroboscopic examination, are likely the cause of her issues. Vocal nodules are benign, typically symmetrical, callous-like growths on both vocal folds that often result from vocal abuse or misuse.  Sevim's profession as a primary school teacher and her extensive voice usage (6 hours a day) indicate a high level of vocal demand. It's not uncommon for individuals in such professions to develop voice disorders due to the strain on their vocal cords. Moreover, her smoking habit could also be exacerbating the condition, as smoking is known to cause irritation and damage to the vocal cords.  Her Voice Handicap Index-10 (VHI-10) score is 26 and Voice-Related Quality of Life (V-RQOL) score is 27, which reflect a significant perceived voice handicap and a poor voice-related quality of life.  Here are some possible recommendations for her:  **1. Voice Therapy:** Sevim should consult a certified Speech-Language Pathologist for a customized voice therapy program. This could include techniques such as vocal hygiene education (to reduce behaviors that are harmful to the vocal cords), relaxation techniques, voice placement exercises, and breath control exercises.  **2. Lifestyle Changes:** She needs to reduce her vocal demand, if possible. This could include using non-verbal cues when teaching or using a voice amplification device. Also, Sevim should consider smoking cessation, as it can improve the health of her vocal cords.  **3. Hydration:** As she is drinking a significant amount of tea and coffee daily, it's crucial to ensure that she maintains good hydration levels. Both coffee and tea can potentially dry out the vocal cords, so increasing water intake could be beneficial.  **4. Medical Treatment:** If voice therapy and lifestyle changes don't result in significant improvements or if her symptoms worsen, Sevim might need to consider surgical intervention for the removal of the vocal nodules. However, this should be considered as a last resort when other conservative measures have been ineffective.  **5. Follow-Up Assessments:** Sevim should have regular follow-up voice assessments to monitor her condition and treatment effectiveness.  Please consider these recommendations in light of her personal circumstances and consult with her healthcare providers for personalized advice. |

**6d) Voice Disorders - Creating Therapy Stimuli**

| **Prompt:**  Create Turkish sample sentences containing /m/ sound for resonant voice therapy. |
| --- |
| **ChatGPT Answer:**  Sure, here are some sample sentences in Turkish for practicing the /m/ sound in resonant voice therapy. Please keep in mind that correct pronunciation might require guidance from a language expert or speech therapist.  1. "Mehmet maymunları çok sever."     (Mehmet loves monkeys very much.)  2. "Annesi mavi bir mont giydi."     (His/Her mother wore a blue coat.)  3. "Kırmızı mumlar masada."     (Red candles are on the table.)  4. "Meyveler mutfakta duruyor."     (Fruits are standing in the kitchen.)  5. "Araba muhteşem manzaraya park etti."     (The car parked with a magnificent view.)  6. "Bana mavi bir mektup yazdı."     (She/He wrote me a blue letter.)  7. "Marmara Denizi'ne bakarken mutlu oluyorum."     (I feel happy when I look at the Sea of Marmara.)  8. "Hem mumları hem de ışıkları seviyorum."     (I love both the candles and the lights.)  9. "Kedimiz Minnoş, mamayı çok seviyor."     (Our cat Minnoş loves cat food very much.)  10. "Müzisyenler müziğiyle meşhur oldu."      (The musicians became famous with their music.)  Remember, the focus here is to practice the /m/ sound, so the sentences are tailored to contain words that have this sound in them. Practice each sentence slowly, and increase your speed as you become more comfortable with the sound. If you're unsure about pronunciation, please consult a Turkish speech therapist or language expert. |

**6e) Voice Disorders - Therapy Planning**

| **Prompt:**  Create a voice therapy plan for the client with a voice disorder, whose information I have given below.  The client’s name is Sevim Uzun. Sevim is 35 years old. Her voice assessment was carried out at Türkiye University Speech and Language Therapy Unit on 29 April 2023. Sevim was assessed by the speech and language therapist Fatma Uslu. She smokes half a pack of cigarettes a day and she does not using alcohol. Sevim has never undergone surgery before and she has no history of allergies. Sevim, who does not have reflux, does not have a chronic disease that requiring regular medication. Sevim uses her voice intensively for about 6 hours a day and she is a primary school teacher. She has complaint of hoarseness, which started 1.5 months ago and progressed. Sevim drinks an average of 5 cups of tea and 2 cups of coffee a day. Sevim's score in the Voice Handicap Index-10 is 26. Her score on the Voice-Related Quality of Life scale is 27.  Sevim has a perceptually hoarse, breathy and rough voice, with a maximum phonation duration of 9 seconds and a s/z ratio of 1.6. Her GRBAS score is 7. Videolaryngostroboscopic examination revealed bilateral nodules in the anterior 1/3 of her vocal folds. |
| --- |
| **ChatGPT Answer:**  Based on the information given about Sevim, her voice disorder appears to be largely related to vocal nodules. Here is a plan for voice therapy for Sevim:  **I. Education (1-2 sessions)**  - Explanation of the vocal mechanism and how nodules develop.  - Discuss the impact of lifestyle habits like smoking and high caffeine intake on vocal health.  - Explain how voice use in her occupation might contribute to her voice issue.  **II. Vocal Hygiene (1-2 sessions)**  - Advise Sevim to reduce smoking or quit entirely.  - Encourage increased hydration and possibly reducing her intake of caffeine.  - Discuss the importance of taking vocal rest periods during her intensive voice use periods.  - Encourage the use of a microphone or other amplification devices during teaching to reduce vocal strain.  **III. Direct Voice Therapy (4-6 weeks)**  **- Breathing exercises:** to improve breath control, support, and reduce strain during phonation. Exercises can include diaphragmatic breathing and pursed lip breathing.  **- Phonatory exercises:** Initiate the use of vocal function exercises to improve the efficiency of her vocal fold vibration and reduce vocal fatigue. This can include exercises such as the pitch glide or sustained phonation exercises.  **- Resonance Therapy:** Use of techniques such as forward focus or resonant voice therapy to create easier voice production, reducing strain on the vocal folds.  **- Voice Rest:** Strategically rest the voice throughout the day, especially during periods of intensive voice use.  **- Elimination of Phonotraumatic Behaviors:** Techniques will be introduced to eliminate any possible abusive vocal behaviors like yelling, frequent throat clearing, etc.  **IV. Follow-up Sessions**  - After completion of the initial therapy, schedule follow-up sessions to monitor progress and to make any necessary adjustments to the therapy.  - If there is no improvement or if Sevim's symptoms worsen, a referral back to the ENT for a possible surgical intervention might be necessary.  **V. Home-Based Maintenance Plan**  - Develop a home-based voice exercise plan for Sevim to follow, including warm-ups and cool-downs for her voice, especially on days with heavy voice use.  - Emphasize the importance of continued good vocal hygiene and encourage lifestyle changes that support this (quitting/reducing smoking, increasing hydration).  **VI. Regular Re-Evaluations**  - Schedule regular re-evaluations of Sevim's voice. Monitor Voice Handicap Index-10 and Voice-Related Quality of Life scores over time, along with perceptual characteristics of the voice and laryngeal imaging as necessary.  Throughout the therapy process, it will be important to provide Sevim with positive reinforcement and validation as she learns to navigate her voice disorder and make changes to her lifestyle and occupational voice use. Therapy will be most successful if it is tailored to Sevim's unique needs and daily routine. |

**6f) Voice Disorders - Creating Education Material**

| **Prompt:**  Create a client education material for clients with voice disorders whose has vocal fold nodules. |
| --- |
| **ChatGPT Answer:**  **Understanding and Managing Vocal Fold Nodules**  **What Are Vocal Fold Nodules?**  Vocal fold nodules, sometimes referred to as "singer's nodules", are noncancerous, callous-like growths that develop on both sides of the vocal cords. They are usually caused by vocal abuse or misuse, like yelling, talking loudly, or singing improperly over extended periods.  These nodules can change your voice, making it sound hoarse, breathy, or rough. Vocal fatigue and a decrease in vocal range may also occur.  **How are Vocal Fold Nodules Diagnosed?**  Your healthcare provider, typically an otolaryngologist, will conduct a thorough examination of your vocal folds using a laryngoscope or a stroboscope. These devices provide a detailed view of your vocal cords and how they're functioning, helping to identify any abnormalities like nodules.  **How Are Vocal Fold Nodules Treated?**  Treatment for vocal fold nodules typically involves a combination of voice therapy and lifestyle changes. Occasionally, if the nodules are large or persistent, surgery may be recommended.  **1. Voice Therapy:** A trained speech-language pathologist will guide you through exercises to improve your vocal techniques and habits. This might involve breath control exercises, vocal hygiene education, and guidance on reducing voice misuse.  **2. Lifestyle Changes:** This can include staying hydrated, avoiding caffeine and alcohol, quitting smoking, resting your voice regularly, and avoiding shouting or straining your voice  **3. Surgery:** This is usually the last resort when conservative methods don't provide relief. However, even after surgery, voice therapy is essential to prevent recurrence.  **Tips for Managing Vocal Fold Nodules**  **1. Stay Hydrated:** Drinking plenty of water keeps your vocal cords moist and reduces the chances of irritation.  **2. Avoid Vocal Strain:** Don't shout or scream, and try not to talk excessively, particularly in loud environments.  **3. Proper Breathing Techniques:** Learn to breathe from your diaphragm instead of your chest to reduce strain on your vocal cords.  **4. Vocal Rest:** Give your voice a break regularly. This is especially important if you use your voice intensively, like teachers, singers, and actors.  **5. Avoid Irritants:** Alcohol, caffeine, and smoking can dry and irritate your vocal cords.  **6. Warm-Up Your Voice:** Just like any other muscles in your body, your vocal cords need to warm up before heavy use.  **7. Mind Your Diet:** Certain foods can cause acid reflux, which can irritate the vocal cords. Spicy food, acidic food, and food high in fat can trigger acid reflux.  **Conclusion**  Remember, everyone's voice is unique, and the management of vocal fold nodules can vary from person to person. It is important to consult with a voice therapist or an otolaryngologist if you suspect you have vocal fold nodules.  In any case, adopting a healthier voice lifestyle can prevent future voice problems and help you maintain a healthy and functioning voice.  *This information is intended to be used as a guide and does not replace professional medical advice. Always consult with a healthcare professional for accurate information.* |

**7a) Swallowing Disorders - Report Writing**

| **Prompt:**  Write a swallowing assessment report according to the information I will give you below.  The client’s name is Mehmet Gedik. Mehmet is 48 years old. Swallowing assessment was carried out at Türkiye University Speech and Language Therapy Unit on 28 April 2023. Mehmet was assessed by the speech and language therapist Fatma Uslu. Mehmet was diagnosed with oral cancer one year ago and he started radiation therapy. The right lower buccal mucosa and the soft tissue of the anterior floor of the mouth with tumor involvement were resected 3 months ago. The radial forearm free flap was used for defects of the oral cavity reconstruction. The oral mechanism exam revealed, weakness of the lips during maximum compression. He complains of choking when swallowing solid foods and liquids. He has limited range of motion of the upper and lower lips on the protrusion. His tongue protrusion, tongue lateralization and maximal jaw opening are restricted. Temporomandibular joint range of motion measured by Therabite was 28 mm. Mehmet was %100 PEG dependent. He was performed 3 oz water test. Mehmet coughed and had difficulty swallowing, drooling, and oral residue. There was no change in his voice quality after the water test. Modified Barium Swallow Study (MBSS) was completed and scored using Penetration-Aspiration Scale (PAS). His PAS score was 5 and his Functional Oral Intake Scale (FOIS) was Level 1. His M. D. Anderson Dysphagia Inventory (MDADI) score is 38 points. |
| --- |
| **ChatGPT Answer:**  **Swallowing Assessment Report**  **Client's Name:** Mehmet Gedik  **Date of Birth:** (Assumed around 1975 based on age given)  **Date of Evaluation:** 28 April 2023  **Assessment Location:** Türkiye University Speech and Language Therapy Unit  **Assessing Therapist:** Fatma Uslu  **Medical History:**  Mehmet was diagnosed with oral cancer approximately one year ago and underwent radiation therapy. The right lower buccal mucosa and the soft tissue of the anterior floor of the mouth were resected due to tumor involvement three months prior to this assessment. A radial forearm free flap procedure was performed to address defects in the oral cavity reconstruction.  **Presenting Symptoms:**  Mehmet has reported difficulties in swallowing both solid foods and liquids, leading to instances of choking. He also experiences drooling and oral residue post-swallow, indicating potential dysphagia. Despite these symptoms, there have been no changes noted in the quality of his voice post water-swallow test. Currently, Mehmet is entirely dependent on percutaneous endoscopic gastrostomy (PEG) for his nutritional needs.  **Oral Mechanism Examination Findings:**  Upon examination, there was noticeable weakness in the lips during maximum compression. The range of motion in both the upper and lower lips was limited during protrusion. Restrictions were also noted in tongue protrusion, tongue lateralization, and maximal jaw opening. Temporomandibular joint range of motion, measured by Therabite, was determined to be 28mm.  **Swallowing Assessment Findings:**  Mehmet was subjected to a 3 oz water test during which he displayed difficulty swallowing, drooling, and oral residue. Despite these signs, there was no alteration in voice quality post water test. A Modified Barium Swallow Study (MBSS) was conducted and scored using the Penetration-Aspiration Scale (PAS). Mehmet's PAS score was 5, indicating there may be material entering the airway with efforts made to eject. The Functional Oral Intake Scale (FOIS) was measured at Level 1, indicative of severe oral intake restriction where the patient is tube dependent with minimal attempts of food or liquid intake. On the M. D. Anderson Dysphagia Inventory (MDADI), Mehmet scored 38 points, indicating a perceived impact on his quality of life due to dysphagia.  **Recommendations:**  Considering the severity of Mehmet's symptoms, the findings from the swallowing assessment, and his current dependence on PEG for nutrition, intensive speech and swallowing therapy is recommended. Further evaluation by an otolaryngologist and a gastroenterologist may be beneficial. Additionally, dietary modifications under the guidance of a dietitian might assist in managing the current swallowing difficulties.  A reassessment should be planned after a few weeks of therapy to monitor progress and adjust the treatment plan as necessary.  **Report Prepared by:** Fatma Uslu  **Date of Report:** 10 July 2023 |

**7b) Swallowing Disorders - Creating Assessment Material**

| **Prompt:**  Create clinical swallowing assessment battery for head-neck cancer patients. |
| --- |
| **ChatGPT Answer:**  **Swallowing Assessment Battery for Head-Neck Cancer Patients**  **1. Patient Interview & Medical History Review:**      - Symptoms review: weight loss, change in diet, coughing, choking, dysphagia (difficulty swallowing)      - Treatment history: surgeries, radiation therapy, chemotherapy      - Health habits: alcohol use, tobacco use  **2. Oral Motor Examination:**      - Examination of oral structures: lips, tongue, palate, dentition      - Function testing: tongue movements, jaw mobility, lip seal  **3. Cranial Nerve Assessment:**      - Cranial nerves that affect swallowing should be assessed including CN V (Trigeminal), VII (Facial), IX (Glossopharyngeal), X (Vagus), XI (Spinal Accessory), and XII (Hypoglossal).  **4. Swallowing Efficiency Testing:**      - Water Swallow Test: measuring the amount and speed of swallowing      - Repetitive Saliva Swallowing Test (RSST): ability to swallow saliva repetitively      - Food and liquid trials: evaluate patient's ability to swallow various consistencies (e.g., puree, soft solid, liquid) safely  **5. Fiberoptic Endoscopic Evaluation of Swallowing (FEES):**      - Direct visualization of the pharyngeal and laryngeal structures during swallowing  **6. Videofluoroscopic Swallow Study (VFSS) or Modified Barium Swallow (MBS):**      - Radiographic assessment of swallowing function to examine the oral, pharyngeal, and esophageal stages of swallowing  **7. Objective Swallowing Measures:**      - Penetration-Aspiration Scale: assess the severity of aspiration/penetration during swallowing      - Temporal Measures: include parameters such as pharyngeal transit time, swallowing reaction time, etc.  **8. Quality of Life Measures:**      - Swallowing Quality of Life Questionnaire (SWAL-QOL): a patient-reported outcome measure  **9. Dietary Assessment:**      - Assessment of current diet and modifications if required      - Nutritional consultation: if required, involvement of a dietitian for assessment and plan  **10. Speech Therapy Consultation:**      - If dysphagia is noted, involvement of a speech therapist for the development of a therapeutic intervention plan  It's important to note that while this is a comprehensive battery, not all parts may be necessary for all patients. The assessment should be tailored to the specific patient's condition, symptoms, and history. |

**7c) Swallowing Disorders - Clinical Decision Support**

| **Prompt:**  Support me in making a clinical decision about the client whose details I have given below.  The client’s name is Mehmet Gedik. Mehmet is 48 years old. Swallowing assessment was carried out at Türkiye University Speech and Language Therapy Unit on 28 April 2023. Mehmet was assessed by the speech and language therapist Fatma Uslu. Mehmet was diagnosed with oral cancer one year ago and he started radiation therapy. The right lower buccal mucosa and the soft tissue of the anterior floor of the mouth with tumor involvement were resected 3 months ago. The radial forearm free flap was used for defects of the oral cavity reconstruction. The oral mechanism exam revealed, weakness of the lips during maximum compression. He complains of choking when swallowing solid foods and liquids. He has limited range of motion of the upper and lower lips on the protrusion. His tongue protrusion, tongue lateralization and maximal jaw opening are restricted. Temporomandibular joint range of motion measured by Therabite was 28 mm. Mehmet was %100 PEG dependent. He was performed 3 oz water test. Mehmet coughed and had difficulty swallowing, drooling, and oral residue. There was no change in his voice quality after the water test. Modified Barium Swallow Study (MBSS) was completed and scored using Penetration-Aspiration Scale (PAS). His PAS score was 5 and his Functional Oral Intake Scale (FOIS) was Level 1. His M. D. Anderson Dysphagia Inventory (MDADI) score is 38 points. |
| --- |
| **ChatGPT Answer:**  Given the details provided about Mehmet Gedik, we can observe that he has significant swallowing difficulties, or dysphagia, following oral cancer resection and reconstruction. He is PEG (Percutaneous Endoscopic Gastrostomy) dependent and chokes when swallowing both solid foods and liquids.  Based on the Modified Barium Swallow Study (MBSS) with a Penetration-Aspiration Scale (PAS) score of 5, it indicates that material is entering his airway, reaching the vocal folds level but not below. This is consistent with his complaint of choking and his poor performance in the 3 oz water test, where he showed coughing, difficulty swallowing, drooling, and oral residue.    His Functional Oral Intake Scale (FOIS) score is Level 1, which is the lowest level, indicating that he is not taking anything by mouth.  The M. D. Anderson Dysphagia Inventory (MDADI) score of 38 points, which is significantly lower than the maximum score of 100, indicates that he is experiencing severe limitations in his daily life due to dysphagia.  Based on these assessments, the following treatment plan can be proposed:  1. Dysphagia Therapy: This can include a range of therapeutic strategies aimed at strengthening the muscles of the oral mechanism, improving range of motion, and swallowing safety. Given his limitation in tongue and lip movements and weakness in lip compression, exercises targeting these areas might be beneficial.  2. Diet Modification: As he currently cannot tolerate solid foods or liquids by mouth, a strict diet modification is necessary to ensure safety and prevent aspiration. This should be done in consultation with a dietitian.  3. Continued use of PEG feeding: Until his swallowing function improves, Mehmet should continue to receive nutritional support via his PEG tube to maintain his overall health.  4. Regular follow-ups with his speech and language therapist to monitor progress and adjust treatment as necessary.  Remember to involve Mehmet in the decision-making process, ensuring he understands the implications of his condition, and potential benefits and limitations of therapy. His motivation and compliance with the therapy will play a crucial role in his recovery.  These suggestions are intended to be supportive, and all decisions should be made in collaboration with Mehmet, his healthcare team, and according to professional ethical guidelines and regulations. |

**7d) Swallowing Disorders - Creating Therapy Stimuli**

| **Prompt:**  Create a monthly swallowing exercises plan according to the information I will give below.  The client’s name is Mehmet Gedik. Mehmet is 48 years old. Swallowing assessment was carried out at Türkiye University Speech and Language Therapy Unit on 28 April 2023. Mehmet was assessed by the speech and language therapist Fatma Uslu. Mehmet was diagnosed with oral cancer one year ago and he started radiation therapy. The right lower buccal mucosa and the soft tissue of the anterior floor of the mouth with tumor involvement were resected 3 months ago. The radial forearm free flap was used for defects of the oral cavity reconstruction. The oral mechanism exam revealed, weakness of the lips during maximum compression. He complains of choking when swallowing solid foods and liquids. He has limited range of motion of the upper and lower lips on the protrusion. His tongue protrusion, tongue lateralization and maximal jaw opening are restricted. Temporomandibular joint range of motion measured by Therabite was 28 mm. Mehmet was %100 PEG dependent. He was performed 3 oz water test. Mehmet coughed and had difficulty swallowing, drooling, and oral residue. There was no change in his voice quality after the water test. Modified Barium Swallow Study (MBSS) was completed and scored using Penetration-Aspiration Scale (PAS). His PAS score was 5 and his Functional Oral Intake Scale (FOIS) was Level 1. His M. D. Anderson Dysphagia Inventory (MDADI) score is 38 points. |
| --- |
| **ChatGPT Answer:**  Given Mehmet's swallowing evaluation results, a swallowing therapy plan focusing on oropharyngeal strength, coordination, and timing exercises would be beneficial. Here's a tentative monthly plan, which can be adjusted based on his progress. It's highly recommended that he performs these exercises under the supervision of a professional.  **Week 1:**  1. Lip press exercise: Press the lips together as firmly as possible, holding for 3 seconds, and then release. Repeat 10 times per session. Do this 3 times a day.  2. Tongue stretches: Stretch the tongue forward towards the nose, then lower lip, then left and right cheeks. Hold each position for a few seconds. Repeat 5 times for each direction, twice a day.  **Week 2:**  1. Continue with week 1 exercises.  2. Therabite exercises: Aim to gradually increase temporomandibular joint range of motion. Use the device for gentle jaw stretches, and repeat 10 times per session. Do this 3 times a day.  **Week 3:**  1. Continue with exercises from weeks 1 and 2.  2. Add Effortful Swallow: Swallow saliva while squeezing all of the swallowing muscles as hard as possible. Repeat 5-10 times per session, 3 times a day.  3. Add Mendelsohn Maneuver: Swallow saliva while consciously holding the larynx at its highest position for 2 seconds during the swallow. Repeat 5-10 times per session, 3 times a day.  **Week 4:**  1. Continue with exercises from previous weeks.  2. Supraglottic swallow: Take a deep breath and hold it. Then swallow while still holding the breath, followed by a cough after the swallow. Repeat this 5-10 times per session, twice a day.  3. Shaker exercise: Lie flat on the back, without a pillow. Lift the head to look at the toes while keeping the shoulders flat on the ground, hold for a few seconds, and then relax. Do this 3 times a day.  Re-assess the swallowing function at the end of the month. Consider a repeat MBSS and/or FOIS score to measure the progress and adjust the exercise plan as needed.  This plan should be followed under the guidance of a speech and language therapist to ensure that the exercises are performed correctly and safely. Always consult the therapist before starting any new exercise or making any changes to the current plan.  **Note:** All exercises may not be suitable for all patients, and the therapist's professional judgment will determine which exercises are most appropriate for Mehmet's unique case. |

**7e) Swallowing Disorders - Therapy Planning**

| **Prompt:**  Create a swallowing therapy plan for the client with a swallowing disorder, whose information I have given below.  The client’s name is Mehmet Gedik. Mehmet is 48 years old. Swallowing assessment was carried out at Türkiye University Speech and Language Therapy Unit on 28 April 2023. Mehmet was assessed by the speech and language therapist Fatma Uslu. Mehmet was diagnosed with oral cancer one year ago and he started radiation therapy. The right lower buccal mucosa and the soft tissue of the anterior floor of the mouth with tumor involvement were resected 3 months ago. The radial forearm free flap was used for defects of the oral cavity reconstruction. The oral mechanism exam revealed, weakness of the lips during maximum compression. He complains of choking when swallowing solid foods and liquids. He has limited range of motion of the upper and lower lips on the protrusion. His tongue protrusion, tongue lateralization and maximal jaw opening are restricted. Temporomandibular joint range of motion measured by Therabite was 28 mm. Mehmet was %100 PEG dependent. He was performed 3 oz water test. Mehmet coughed and had difficulty swallowing, drooling, and oral residue. There was no change in his voice quality after the water test. Modified Barium Swallow Study (MBSS) was completed and scored using Penetration-Aspiration Scale (PAS). His PAS score was 5 and his Functional Oral Intake Scale (FOIS) was Level 1. His M. D. Anderson Dysphagia Inventory (MDADI) score is 38 points. |
| --- |
| **ChatGPT Answer:**  Based on the information provided, Mehmet Gedik has been diagnosed with a significant swallowing disorder, or dysphagia, following treatment for oral cancer. The treatment included surgery and radiation, which have resulted in muscular weakness and decreased range of motion impacting his oral structures, making it difficult for him to swallow both solids and liquids. Consequently, his overall oral intake has been severely compromised.  Considering the reported symptoms, a multi-faceted approach to swallowing therapy would be recommended to help him regain some swallowing function and improve his quality of life. Here is a suggested plan:  **1. Oral Motor Exercises:** These will focus on improving the strength, range, and coordination of the muscles involved in swallowing. Specific exercises would include lip press exercises, tongue exercises (such as tongue press, tongue lateralization, and tongue stretching), and jaw exercises. These should be performed daily as prescribed by the therapist.  **2. Swallowing Exercises:** These are designed to improve the mechanics of swallowing. Exercises may include effortful swallow, supraglottic swallow, and Mendelsohn maneuver. These techniques can help improve bolus control and enhance the timing and strength of the swallow.  **3. Therabite Exercises:** Given the temporomandibular joint range of motion measurement of 28 mm, use of a Therabite device could be beneficial to improve jaw opening and overall mouth mobility. The Therabite exercise regimen should be followed strictly as per the therapist's recommendation.  **4. Nutritional Management**: Given that Mehmet is currently 100% PEG dependent, gradual reintroduction of oral feeding should be considered, depending on his progress with the swallowing therapy. A dietitian should be involved to help manage his nutritional needs and help transition from PEG feeding to oral feeding.  **5. Behavioral Strategies:** These include alterations to eating positions, use of particular swallowing techniques, and modifications to the texture and consistency of food and liquid. For example, starting with pureed foods or thickened liquids might be helpful initially, and then gradually introducing foods of different consistencies as swallowing function improves.  **6. Hydration and Oral Hygiene:** Regular oral hygiene should be maintained to prevent infections and complications associated with dysphagia and PEG feeding.  **7. Regular Assessments:** Regular reassessment of Mehmet's swallowing function using MBSS, PAS, FOIS, and MDADI scores would be crucial to monitor his progress and adjust treatment as necessary.  **8. Psychological Support:** Given the nature of his condition, psychological support may also be beneficial, as dysphagia can significantly impact a person's emotional well-being.    This plan is a general outline, and the specifics should be tailored to Mehmet's individual condition and needs. It is also crucial that the therapy is carried out under the supervision of a trained speech and language therapist. |

**7f) Swallowing Disorders - Creating Education Material**

| **Prompt:**  Create a client education material for clients with swallowing disorders in head and neck cancers. |
| --- |
| **ChatGPT Answer:**  **Understanding Swallowing Disorders in Head and Neck Cancers**  **Introduction**  When you're diagnosed with a head or neck cancer, you're likely to face a number of challenges related to the disease and its treatment. One such challenge may be a swallowing disorder, also known as dysphagia. The ability to swallow may be affected by the tumor itself or as a side effect of treatments like surgery, radiation, or chemotherapy. Here's some information to help you understand and cope with dysphagia.  **Swallowing and its Importance**  Swallowing, a complex process that involves over 50 pairs of muscles and many nerves, allows us to eat, drink, and take medications. A disorder in this process can lead to significant health issues, including dehydration, malnutrition, and even aspiration pneumonia (where food or liquid enters the lungs, leading to infection).  **Why Dysphagia Occurs in Head and Neck Cancer**  In head and neck cancers, dysphagia can occur due to:  - The tumor interfering with the normal swallowing mechanism.  - Surgery to remove the tumor that affects muscles and structures involved in swallowing.  - Radiation therapy that can cause inflammation, fibrosis, or xerostomia (dry mouth), impacting the swallowing process.  - Chemotherapy leading to mouth sores and inflammation of the oral cavity and throat.  **Identifying Dysphagia**  Signs of a swallowing disorder may include:  - Difficulty starting a swallow.  - Choking or coughing during or after eating or drinking.  - Feeling like food is stuck in your throat or chest.  - Drooling or spilling food out of the mouth.  - Unexplained weight loss.  - Changes in voice after eating or drinking.  **Managing Dysphagia**  Management and treatment strategies for dysphagia can be multi-faceted, including:  **- Speech and Language Therapist (SLT):** SLTs can teach you exercises to strengthen the muscles involved in swallowing, provide tips to make swallowing safer and easier, and help to adjust your diet as needed.  **- Nutritional support:** Dietitians can assist with modifying food textures and consistencies, ensuring you are getting necessary nutrients while minimizing difficulties with swallowing.  **- Medical interventions:** Medications to manage pain or reduce inflammation may be prescribed. In some cases, surgical or endoscopic interventions might be necessary to improve swallowing function.  **- Mindful Eating:** Taking smaller bites, eating slowly, and concentrating on the act of swallowing can often help manage symptoms.  **- Hydration:** Stay hydrated but be careful with thin liquids which can be hard to control and may increase the risk of aspiration.  **Conclusion**  Dealing with dysphagia can be challenging, but remember that you're not alone. A multidisciplinary team of healthcare professionals is available to provide support and treatment. Reach out to your healthcare provider if you are experiencing any difficulties swallowing or if you notice any changes in your ability to eat or drink.  This information is meant to be educational and should not replace the advice from your healthcare provider. Always consult your healthcare provider or therapist for personalized advice.  *Disclaimer: This material is meant for educational purposes only and does not constitute medical advice. Always consult with a healthcare professional for proper diagnosis and treatment.* |
